# Isolation, characterization, and biological evaluation of endophytic fungi from *Phragmites australis*: experimental and computational insights

**DOI:** 10.3389/fmolb.2025.1713876

**Published:** 2026-01-09

**Authors:** Dina Mahfouz Eskander, Mohamed E. El Awady, Mohamed Ali, Asmaa M. Fahim, Ahmed A. Hamed, Basel Sitohy

**Affiliations:** 1 Chemistry of Natural Compounds Department, National Research Centre, Cairo, Egypt; 2 Microbial Biotechnology Department, National Research Centre, Cairo, Egypt; 3 Biochemistry Department, Faculty of Science, Zagazig university, Zagazig, Egypt; 4 Department of Green Chemistry, National Research Centre, Cairo, Egypt; 5 Microbial Chemistry Department, National Research Centre, Cairo, Egypt; 6 Department of Clinical Microbiology, Infection, and Immunology, Umeå University, Umeå, Sweden; 7 Department of Diagnostics and Intervention, Oncology, Umeå University, Umeå, Sweden

**Keywords:** endophytic fungi, secondary metabolites, biological activities, ADMET analysis, molecular docking, molecular dynamics, density function theory studies

## Abstract

Endophytic fungi are an uncharted source of bioactive metabolites with varied therapeutic characteristics. In this research, an endophytic *Aspergillus* sp. (HAG1) was collected from *Phragmites australis* and identified using morphological and molecular methods. The large-scale fermentation, chromatographic purification, and spectroscopic approaches (FT-IR, UV-Vis, ^1^H NMR, and ESI-MS) resulted in the identification of three metabolites: vaccenic acid (**C1**), pipericine (**C2**), and guaiacylglycerol (**C3**). Of these, **C3** is reported here for the first time as an endophyte-derived metabolite from *P. australis*. All the metabolites exhibited significant antioxidant, antibacterial, antibiofilm, and anti-inflammatory activity. The activities of **C3** were the most effective in DPPH and ABTS scavenging, COX-1/COX-2 inhibition, and suppression of biofilm for bacteria, although **C3** was inactive against acetylcholinesterase activity. Molecular docking and molecular dynamics (MD) simulations underscored a favorable binding with a high binding conformation stability of **C3** for antioxidant (1DGF), anti-inflammatory (3NLO), and antibiofilm (5TZ1) targets. In addition, density function theory (DFT) calculations delivered insights regarding electronic structure, explaining observed reactivity and hydrogen bonding ability. Moreover, ADMET predictions indicated that **C3** has favorable solubility, metabolic stability, and low toxicity when compared to **C1** and **C2**.

## 1 Introduction

Endophytes are microorganisms that inhabit plant tissues without causing observable symptoms of illness (Wani et al., 2015). These internal symbionts, especially fungi, play a crucial role in plant health by filling ecological niches that might otherwise be occupied by pathogens, outcompeting them for nutrition, producing antimicrobial compounds, and enhancing plant defense mechanisms. Endophytic fungi may promote plant growth by secreting growth regulators and enhancing plant resilience to environmental stresses. Certain organisms also synthesize chemicals that function as natural insect repellents (Lubna et al., 2018; Badawy et al., 2021; Abdelaziz et al., 2022).

Plants harbor a varied assemblage of microorganisms, including bacteria and fungi, collectively known as the plant microbiome. Endophytic fungi represent a notably abundant and diverse category, estimated to include approximately one million distinct species (Nisa et al., 2015; Dhyani et al., 2019). Recently, there has been an increased emphasis on isolating and characterizing these fungi, motivated by their extensive array of natural compounds. Endophytes have more diverse metabolic characteristics than several free-living soil fungi (Dhayanithy et al., 2019). Interest in endophytes as a source of novel therapeutics intensified after significant discoveries, including the anticancer agent paclitaxel (Taxol), first isolated from the endophyte *Taxomyces andreanae* in the early 1990s, and penicillin, extracted from *Penicillium notatum* by Alexander Fleming in 1928. Paclitaxel was first extracted from the bark of *Taxus brevifolia* and subsequently identified as being synthesized by related endophytes, including *Taxomyces andreanae* and *Pestalotiopsis microspora* (Strobel et al., 1996).

These advancements highlighted the unexploited capacity of endophytes as sources of pharmacologically significant substances. Endophytic fungi have produced many antibacterial compounds that are effective against drug-resistant infections. The profile and yield of these bioactives are affected by parameters like the collection season, ecological context (e.g., salty areas, high altitudes, rainforests, and dry zones), sampled plant component (roots, leaves, and seeds), and host plant type (angiosperms vs. gymnosperms) (Gupta et al., 2020).

Supplementary variables such as sample health status, soil attributes (pH, type, and microbial composition), and climatic factors (temperature, humidity, and light) also influence endophyte diversity and metabolite synthesis (Suryanarayanan et al., 2005). Prominent bioactive chemicals extracted from endophytic *Aspergillus fumigatus* include linoleic acid, R-glycerol monolinoleate, fumiquinazoline derivatives, cerebroside A, and pyrazoline-3-one trimer, among others. These fungi have shown prolific production of several chemical scaffolds, including alkaloids, terpenoids, and polyphenolics, such as hexadecanoic acid esters, bisabolol oxide B, glycidyl derivatives, and linoleoyl chloride (Sharaf et al., 2022). Crude extracts from *Aspergillus* species have substantial antibacterial and antioxidant properties, owing to their synthesis of strong secondary metabolites (Sharaf et al., 2022).

Certain chemicals may derive from the fungus or from horizontal gene transfer activities affecting the host plant genome (Hussain et al., 2012). The isolation of paclitaxel from *T. andreanae*, an endophyte of *T. brevifolia*, is one of the most well-reported instances, representing significant progress in cancer therapies (Kaul et al., 2012). Despite the identification of many plant-derived sources of paclitaxel, a cost-effective synthetic method remains unattainable (Nisa et al., 2015). Recently, species like *Alternaria* have been identified as producers of metabolites exhibiting cytotoxic, anti-trypanosomal, and anti-leishmanial properties. Similarly, *Berkleasmium* species have produced chemicals like diepoxin and palmarumycin derivatives that have significant antifungal properties (Shan et al., 2014).

Endophytic fungi are increasingly acknowledged for their biosynthetic potential across several sectors, including medicines, agriculture, cosmetics, and food. Their secondary metabolites include many functional groups, such as alkaloids, terpenoids, polyketides, peptides, xanthones, furandiones, and depsipeptides, which enhance their extensive bioactivity (Sharaf et al., 2022).

This study was undertaken to isolate and characterize endophytic fungi associated with *Phragmites australis*, as well as to examine their ability to produce biologically active secondary metabolites. More specifically, the objectives of the study were to (i) identify the endophytic strain that was isolated by morphological and molecular methods; (ii) purify and provide structural characterization of its major metabolites by means of a suite of spectroscopic and spectrometric approaches (^1^H NMR, FT-IR, and UV spectrum) and (iii) assess the antioxidant, antimicrobial, antibiofilm, and anti-inflammatory bioactivities of the purified compounds in *in vitro* and *in silico* studies. Compound **C3** (guaiacylglycerol) received special focus, as it was the most active metabolite with multitarget biological activity. Therefore, additional computational studies such as molecular docking, molecular dynamics (MD) simulations, ADMET predictions, and density functional theory (DFT) calculations were performed to help rationalize its structural, electronic, and pharmacokinetic properties. This work collectively aims to establish a mechanistic and predictive framework that links the chemical reactivity of endophyte-derived metabolites, with a focus on **C3**, to the biological effects observed *in vitro*, contributing to the development of lead scaffolds for future therapeutic development.

Although endophytes of *P. australis* and their metabolomes have been investigated, none of the previous studies have reported vaccenic acid, pipericine/piperic acid, or guaiacylglycerol as metabolites derived from endophytic fungi associated with *P. australis*. Therefore, this study provides the first report of the specific metabolites from *P. australis* endophytes and offers the first experimental testing of guaiacylglycerol (**C3**) in antibiofilm and COX-1/COX-2 anti-inflammatory assays, with AChE screening (negative at all concentrations tested up to 100 μg/mL) to provide additional information on target selectivity (Schroeder et al., 2020).

## 2 Experimental section

### 2.1 Instruments

#### 2.1.1 NMR analysis

NMR spectra were recorded on a JEOL Ex-500 spectrometer. ESI-MS was recorded on a Waters-Micromass Quattro Premier Triple Quadrupole mass spectrometer. Column chromatography was carried out on silica gel F254 (Merck) in glass columns. Sephadex LH-20 column chromatography was used for purification. Thin-layer chromatography (TLC) was performed with silica gel 60 GF254 plates (Merck, Darmstadt, Germany), and then the plates were visualized by UV light and spraying vanillin in H_2_SO_4_.

##### 2.1.1.1 Vaccenic acid (**C1**)

White amorphous powder, ESI-MS m/z 283.461 [M + H]^+^ molecular formula C_18_H_34_O_2_. ^1^H NMR (400 MHz, CDCl_3_, δ ppm): δ 0.91 (t, J = 6.8 Hz, 3H, –CH_3_), δ 1.31–1.55 (m, 20H, –CH_2_– chain), 1.91 (q, J = 7.2 Hz, 2H, –CH_2_–CH_2_–CH_3_), 2.05–2.47 (m, 2H, CH_2_ adjacent to C=O), 2.60–2.73 (m, 2H, allylic CH_2_), 3.33–3.65 (m, 2H, –CH_2_–O–), 4.16–4.23 (m, 1H, CH–OH), 5.31 (br s, 1H, olefinic = CH–), 8.46 (s, 1H, amide–NH–).

##### 2.1.1.2 Pipericine (**C2**)

Oily, ESI-MS m/z 336.32 [M]^+^ molecular formula C_22_H_41_NO. ^**1**^H NMR (400 MHz, CDCl_3_, δ/ppm): δ 0.97 (t, 9H, J = 7.2 Hz, CH_3_), 1.31–1.87 (m, 22H, (CH_2_)_3_- backbone), 2.15 (t, 2H, J = 7.4 Hz, CH_2_–CO–), 2.50 (s, 2H, –CH_2_–), 2.75 (dd, 1H, J = 10.4, 4.8 Hz, CH–O–), 4.89 (s, 1H, OH), 6.76 (d, 1H, J = 8.0 Hz, CH =), 7.65 (d, 1H, J = 8.2 Hz, CH =), 7.73 (s, 1H, CH =), 8.58 (s, 1H, NH).

##### 2.1.1.3 Guaiacylglycerol (**C3**)

Colorless amorphous powder, ESI-MS m/z 214.2 [M]^+^ molecular formula C_10_H_14_O_5_. (3-(4-Hydroxy-3-methoxyphenyl)-1,2-propanediol,threo). ^1^H NMR (400 MHz, DMSO-d_6_, δ ppm, J in Hz): 9.10 (s, 1H, Ar-OH), 6.90 (d, J = 1.8, 1H, H-2), 6.78 (d, J = 8.0, 1H, H-5), 6.73 (dd, J = 8.0, 1.8, 1H, H-6), 4.86 (d, J = 5.6, 1H, HO-7), 4.62 (dd, J = 6.3, 5.6, 1H, H-7, CH–OH), 4.40 (d, J = 5.3, 1H, HO-8), 3.92 (dd, J = 7.6, 5.3, 1H, H-8, CH–OH), 3.83 (s, 3H, OMe), 3.72 (dd, J = 11.5, 6.6, 2H, H-9a, CH_2_OH), 3.57 (dd, J = 11.5, 5.3, 1H, H-9b, CH_2_OH).

##### 2.1.1.4 FT-IR analysis

Using a KBr pellet method on Shimadzu IRTracer-100 Spectralizer, the Fourier transform infrared (FT-IR) spectra of compounds **C1**, **C2**, and **C3** were obtained in a region of 4,000–400 cm^−1^. Approximately 1 mg of each sample was finely ground, mixed with spectroscopic-grade KBr (100 mg), and pressed into transparent pellets under vacuum. A resolution of 4 cm^−1^ was used to collect the FT-IR spectra, and each spectrum was averaged over 32 scans to reduce background noise. The data obtained were analyzed in OriginPro, which allowed for a comparative spectral overlay to observe characteristic vibrational modes of the functional groups and band shifts in the three compounds.

##### 2.1.1.5 UV-Visible

The UV-Vis absorption spectra of the synthesized compounds **C1**, **C2**, and **C3** were recorded in methanolic solution at room temperature using a double-beam UV-Visible spectrophotometer within the wavelength range 190–400 nm. The solutions were freshly prepared with an approximate concentration of 1 × 10^−5^ M, ensuring transparency and avoiding aggregation effects. Quartz cuvettes of 1 cm path length were employed, and the baseline was corrected with pure methanol as a reference. The spectra were collected under identical instrumental parameters, including a scanning speed of 200 nm min^−1^ and a spectral resolution of 1 nm.

### 2.2 Collection of plant samples

The plant *Phragmites australis* was collected from El-Beheira, Egypt, in 2024, and identified based on morphological features by the Microbial Chemistry Department, National Research Centre, Egypt. The plant specimen was brought to the laboratory, given a code, photographed, and stored in a refrigerated environment at 5 °C until fungal strains were identified.

### 2.3 Isolation of plant-associated fungus

**FIGURE 1:**
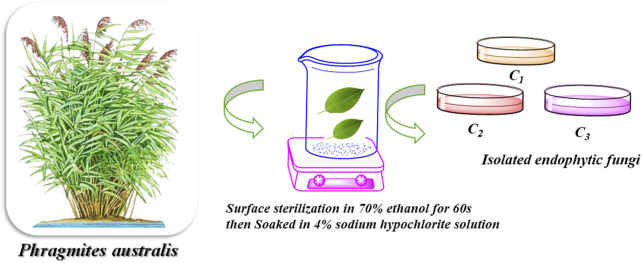
Schematic representation of the procedure for isolation of endophytic fungi from the collected plant tissues. The workflow outlines the steps involved in the order in which they are performed: surface sterilization, inoculation on selective media, incubation, and colony purification for subsequent morphological and molecular identification.

Isolation of the endophytic fungi from the plant was carried out via surface sterilization. In brief, plant leaves were treated with 75% ethanol for 30 s, followed by 0.5% sodium hypochlorite solution for 30 s. Then, leaves were rinsed three times with sterilized distilled water and dried under aseptic conditions. The leaves were then cut and incubated in potato dextrose agar medium at 30 °C for 5 days. After the specified period of incubation, the fungal colonies were moved to potato dextrose agar medium plates and cultivated under the same conditions displayed in Figure 1.

### 2.4 Morphological identification

The most potent fungal isolates were phenotypically characterized by growing them on potato dextrose agar medium plates for 10 days. The study focused on analyzing colony morphology, encompassing features including color, shape, and medium pigmentation. Microscopic detection was undertaken to determine the mycelium and spores produced (Klich, 1988).

### 2.5 Genetic identification of the isolated fungus

Genetic identification of the isolated fungal isolate was performed by genomic DNA extraction using the Qiagen DNeasy Mini Kit. Amplification reactions for fungal 18S were conducted with two primers, ITS1 (5′-TCCGTAGGTGAACCTGCG-3′) and ITS4 (5′-TCCTCCGCTTATTGATATGC-3'. The PCR products were sent to two commercial sequencing providers, SolGent and Macrogen, located in South Korea. The sequences were examined utilizing the BLASTN web platform (https://blast.ncbi.nlm.nih.gov/Blast.cgi, accessed December 2023) to evaluate their similarity and shared lineage with the target gene sequences in the NCBI database. The phylogenetic tree was constructed utilizing the maximum likelihood (ML) method with the assistance of MEGA X software (Blunt et al., 2018).

### 2.6 Fermentation and preparation of large crude extract

Preparation of the large crude extract of the obtained fungi was carried out by growing the pure fungal colony on rice medium for 15 days at 30 °C. After incubation, the rice culture was extracted using ethyl acetate. The extract was evaporated under reduced pressure to yield 15 g of crude extract. A portion of this extract was subjected to column chromatography for compound isolation, and the remaining extract was stored for biological study.

### 2.7 Isolation of the compounds

The crude extract was subjected to silica gel column chromatography, eluted with an n-hexane and ethyl acetate step gradient, starting with 100% n-hexane and increasing polarity by increasing ethyl acetate concentration up to 100%. Elutes were monitored by TLC, UV light, and spraying with a 1% vanillin/5% H_2_SO_4_/EtOH reagent. Similar fractions were combined based on TLC profiles, giving three major fractions (F1–F3), which were eluted by different concentrations of ethyl acetate in hexane (Table 1). F1 was purified by TLC on a silica gel “G” plate using hexane/ethyl acetate (9:1) as a developing system, giving compound 1 (**C1**). F2 was purified using hexane/ethyl acetate (8:2) as a developing system, giving compound 2 (**C2**). F3 was purified on a Sephadex LH-20 column with ethyl acetate/hexane (7:3) to afford compound 3 (**C3**). The compounds were identified with their physical and spectroscopic data and by comparing data with reported literature.

**TABLE 1:** Different ethyl acetate concentrations.

Fractions no	Different ethyl acetate concentrations %
F1	10
F2	30
F3	60

### 2.8 Biological evaluation

#### 2.8.1 Antioxidant activities

##### 2.8.1.1 DPPH radical scavenging activity

The free radical scavenging activity of the sample was examined using 1,1-diphenyl-2-picryl-hydrazil (DPPH^•^) according to the method of Ibrahim et al. (2021). Successive concentration (2–10 μg/mL) from **C1**, **C2**, **C3**, and standard materials (ascorbic acid) were used for the determination of DPPH radical scavenging activity. The absorbance was read at 517 nm in a spectrophotometer. Activity was determined according to the following Equation 1:

$$DPPH\;Scavening\;activity\;\left(\%\right)=\left[\left(A\;control-A\;sample\right)÷A\;control\right]\times 100.$$ (1)

##### 2.8.1.2 ABTS radical cation scavenging activity

The approach was outlined by Miller and Rice-Evans (1997). The ABTS radical cation scavenging activity of samples and the positive control (ascorbic acid) was evaluated at various concentrations between 2 μg/mL and 10 μg/mL. The absorbance at 734 nm was quantified to indicate the ABTS radical cation scavenging activity and subsequently computed as follows in Equation 2:

$$ABTS\;Scavening\;activity\;\left(\%\right)=\left[\left(A\;control-A\;sample\right)÷A\;control\right]\times 100.$$ (2)

##### 2.8.1.3 Ferrous ion (Fe^2+^) chelating capacity

The Fe^2+^ chelation capacity of the sample was assessed using the methodology of Dinis et al. (1994). **C1**, **C2**, and **C3** were compared to a standard chemical, ascorbic acid, under identical conditions. Each quantity was supplemented with 0.05 mL of 2 mM FeCl_2_. The reaction commenced with the addition of 5 mM ferrozine (0.2 mL), followed by vigorous shaking and a 10-min standing period at room temperature. The control accurately contained FeCl_2_ and ferrozine. The reaction’s absorbance was measured at 562 nm using a spectrophotometer.

##### 2.8.1.4 Lipid peroxidation in ammonium thiocyanate medium

The capacity of the samples **C1**, **C2**, and **C3** to suppress lipid peroxidation was assessed using the method of Gülçin et al. (2004), compared to ascorbic acid for evaluation purposes. The peroxide concentration was assessed by measuring the absorbance at 500 nm. The percentage inhibition of lipid peroxidation was determined using the following Equation 3:

$$Lipid\;peroxidation\;inhibition\;\left(\%\right)=\left[\left(A\;control-A\;sample\right)÷A\;control\right]\times 100.$$ (3)

##### 2.8.1.5 Nitric oxide radical scavenging activity

The nitric oxide (NO^•^) radical scavenging ability of the investigated compounds (**C1**, **C2**, **C3**, and ascorbic acid) was assessed using sodium nitroprusside (SNP). Nitric oxide (NO) was created from SNP in aqueous solution at physiological pH, resulting in the formation of nitrite ions, which were quantified using the Griess reagent (Balakrishnan et al., 2009). The absorbance of these solutions was quantified at 540 nm relative to the equivalent blank solution.

#### 2.8.2 Evaluation of anti-inflammatory activity

##### 2.8.2.1 *In vitro* cyclooxygenase enzyme (COX-1 and COX-2) inhibition assay

The cyclooxygenase inhibitory efficacy of **C1**, **C2,** and **C3** was assessed using a modified method of Larsen et al. (1996), and celecoxib was used as the standard compound. The concentrations of the tested materials were 0.625, 1.25, 2.5, 5, 10, and 20 μg/mL for COX-1 and COX-2 analyses. Leuco-2,7-dichlorofluorescein diacetate (5 mg) underwent hydrolysis at room temperature in 1 M NaOH (50 μL) for 10 min. Subsequently, 1 M HCl (30 μL) was introduced to neutralize the excess NaOH, after which the resulting 1-DCF was diluted in 0.1 M Tris buffer, pH 8. The cyclooxygenase enzyme (COX-1 or COX-2) was diluted in 0.1 M Tris buffer at pH 8, resulting in a known aliquot that produced an absorbance change of 0.05 per minute in the test reaction. Test samples, or an equivalent volume of methanol (20 μL), were pre-incubated with the enzyme at room temperature for 5 min in the presence of hematin. To initiate the reaction, premixed phenol, 1-DCF, and arachidonic acid were incorporated into the enzyme mixture, resulting in a final reaction composition of arachidonic acid (50 μM), phenol (500 μM), 1-DCF (20 μM), and hematin (1 μM) in a total volume of 1 mL of 0.1 M Tris buffer at pH 8. The reaction was monitored spectrophotometrically for 1 min at a wavelength of 502 nm. A blank reaction mixture was analyzed in the spectrophotometer reference cell alongside each test reaction to control for any non-enzymatic activity related to the test sample. This blank comprised the reaction mixture devoid of enzyme addition. The percentage of COX inhibition was assessed as follows Equation 4:

$$COX\;inhibition\;activity=\left(1-As÷Ac\right)\times 100.$$ (4)

#### 2.8.3 Antimicrobial properties

To investigate the antibacterial effectiveness of different compounds (**C1, C2,** and **C3**), experiments were conducted in flat polystyrene 96-well plates. A 10 µL aliquot of each of the sample extracts (final concentration of 50 μg/mL) was introduced to 80 µL of lysogeny broth (LB broth), followed by 10 µL of isolated bacteria in suspension (log phase), and then incubated at 37 °C for the entire night. *Bacillus subtilis* ATCC 6633 and *Staphylococcus aureus* ATCC 6538-P are Gram-positive bacteria. *Escherichia coli* ATCC 25922 and *Pseudomonas aeruginosa* ATCC 27853, as Gram-negative bacteria, and fungi (*Aspergillus niger* NRRL A-326) served as testing organisms. The absorbance was calculated as a mean standard deviation (SD) in a SPECTROstar Nano Microplate Reader (BMG Labtech GmbH, Allmendgrun, Germany) after approximately 20 h at OD_600_ (Hamed et al., 2020). Minimum inhibitory concentration (MIC) was determined by using different concentrations of **C1**, **C2**, and **C3** (2.5–50 μg/mL).

#### 2.8.4 Antibiofilm activity

An MTP assay was conducted to assess the antibiofilm activity of the purified compound against four clinical microorganisms: *P. aeruginosa* ATCC 27853, *S. aureus* ATCC 6538-P, *E. coli* ATCC 25955, and *B. subtilis* ATCC 6633. The experiment involved inoculating sterile 96-well plates overnight with bacterial suspensions in nutrient-rich broth and introducing the chemical at a concentration of 50 μg/mL. Wells were rinsed with PBS to remove planktonic cells following 24 h of biofilm development at 37 °C and subsequently stained with a 0.1% crystal violet solution for 15 min. Excess stain was removed, and wells were washed and air-dried prior to solubilizing the dye with ethanol. Biofilm inhibition was evaluated using optical density (OD) at 570 nm (Abdellatief et al., 2025). Minimum inhibitory concentration (MIC) was determined by using different concentrations of **C1**, **C2**, and **C3** (2.5–50 μg/mL).

#### 2.8.5 Acetylcholinesterase inhibition efficacy assay

The enzymatic activity was assessed using a modified version of the method outlined by Ingkaninan et al. (2003). A 500 μL aliquot of DTNB (3 mM), 100 μL of AChI (15 mM), 275 μL of Tris-HCl buffer (50 mM, pH 8), and 100 μL of sample at varying concentrations (12.5, 25, 50, and 100 μg/mL) were combined in a 1 mL solution, which served as the blank. In the reaction, 25 μL of buffer was substituted with an equal volume of an enzyme solution at a concentration of 0.28 Uml^−1^. The reaction was observed for 5 min at a wavelength of 405 nm. The reaction velocities were calculated. Enzyme activity was determined as a percentage of the velocities relative to the assay conducted with buffer in place of the tested inhibitor sample. Inhibitory activity was determined by subtracting the percentage of enzyme activity from 100. The data presented are the average of three replicates.

### 2.9 Statistical analysis

All experiments were performed in triplicate, and the findings are reported as the mean ± standard deviation. One-way analysis of variance (ANOVA) was employed for data analysis. The significance was assessed based on the p-value; statistical analysis was carried out using GraphPad Prism 10.5 software.

### 2.10 Docking simulation

Vaccenic acid, pipericine, and guaiacylglycerol were molecularly docked using the Molecular Operating Environment (MOE) program. The Discovery Studio Client (version 4.2) was utilized to locate it (Vilar et al., 2008; Jejurikar and Rohane, 2021). The Confirmation Examination module of AutoDock Vina was utilized to reduce the energy of the acquired conformations after conducting a thorough conformational analysis to an RMS gradient of 0.01, and a molecular dynamics simulation of these metal complexes was made through GROMACS (Van Der Spoel et al., 2005) in water solvent with AMBER/CHARMM with metal-specific parameters at 300 K. The compounds were docked with the twinned 3.35A structure of S. aureus Gyrase complex with ciprofloxacin and DNA, PDB ID: 2XCT (Bax et al., 2010), *Salmonella typhi* OmpF complex with ciprofloxacin PDB ID: 4KRA (Akshay et al., 2023), the human erythrocyte catalase (PDB ID:1DGF) (Putnam et al., 2000), PDB ID: 3QFA (crystal structure of the human thioredoxin reductase–thioredoxin complex) (Fritz-Wolf et al., 2011), the crystal structure of sterol 14-alpha demethylase (CYP51) from *Candida albicans* in complex with the tetrazole-based antifungal drug candidate VT1161 (VT1) (PDB ID: 5TZ1) (Hargrove et al., 2017), mutant P44S M296I of foot-and-mouth disease virus RNA-dependent RNA polymerase (PDB ID: 3Nl0) (Agudo et al., 2010), and the crystal structure of human acetylcholinesterase (PDB ID: 4PQE) (Dym et al., 2015). Ten distributed docking simulations were run with the default parameters. Conformations were made based on the overall data organization, the E conformation, and the correct placement of relevant amino acids in the binding pocket of each protein (Fahim, 2024). The following identifiers were used to obtain all protein crystal structures used in this study from the RCSB Protein Data Bank: 1DGF and 3QFA (antioxidant), 3NL0 (anti-inflammatory), 2XCT and 3KRA (antimicrobial), 5TZI (CYP51 antibiofilm), and 4PQE (acetylcholinesterase).

### 2.11 DFT investigation

Utilizing the Gaussian 09W program, the theoretical investigation was carried out through (DFT/B3LYP/6–311 basis set level using the Berny method (Fahim and Farag, 2020; Fahim and Magd, 2021). No symmetry constraints were applied during the geometry optimization. The wide-ranging vibrational modes were assigned using the potential energy distribution (PED) determined by the vibrational energy distribution analysis (VEDA) program (Mahmoud and Fahim, 2025).

### 2.12 Clarifying the biological evaluation aim

Biological and computational assays were structured independently but synergistically to better encompass the pharmacological efficacy of the isolated metabolites from *Aspergillus* sp. HAG1. Each experimental biological assay was chosen to evaluate an individual pharmacological outcome. Antioxidant and anti-inflammatory assays evaluated redox-modulatory and COX-mediated mechanisms. Antimicrobial and antibiofilm assays evaluated ecological and anti-infective properties. Acetylcholinesterase inhibition was included to assess potential neurological activity as a representative neuroenzyme screening. Correspondingly, the molecular docking studies were positioned to correspond to each biological outcome. Specifically, PDB ID: 1DGF and 3QFA (antioxidants), 3NL0 (anti-inflammation), 2XCT and 3KRA (antimicrobial), 5TZI (antibiofilm), and 4PQE (acetylcholinesterase) proteins were selected to represent diverse potential pharmacological activities, as intended in the biological studies above. Lastly, the DFT and ADMET analyses were conducted to characterize the electronic, structural, and pharmacokinetic parameters of the isolated compounds, leading to a theoretical biological evaluation that corresponds with the experimental observations throughout this work.

## 3 Results

### 3.1 Sample collection

*Phragmites australis* was collected from El-Beheira, Egypt. Isolation of endophytic fungi was carried out, and the obtained fungus was coded as HAG1. The obtained strain was stored in a culture collection at 4 °C until use.

### 3.2 Genetic identification of the isolated fungal strains

**FIGURE 2:**
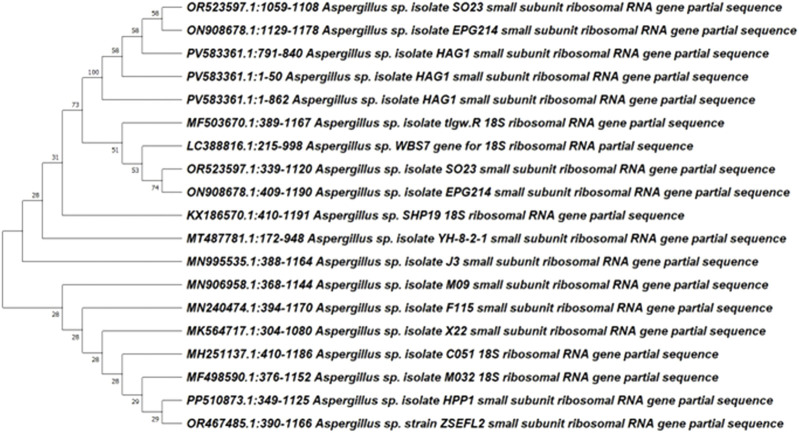
Neighbor-joining phylogenetic tree of the partial sequence of 18S rRNA of the local isolate *Aspergillus* sp. HAG1 with respect to closely related sequences available in GenBank databases.

The isolated fungus HAG1 was identified by conducting a sequencing analysis of the 18S rRNA gene, following a preliminary investigation of the isolated fungus HAG1. The DNA of the fungal sample was obtained, identified, and compared to existing sequences in the GenBank database using the BLAST tool (http://www.blast.ncbi.nlm.nih.gov/Blast, accessed on 2 June 2025). This was done to determine the similarity score and statistical significance of the matches. The isolate’s 18S rRNA gene sequences were found to be identical to those of *Aspergillus* sp. HAG1, showing 100% identity, according to the data. The strain was identified and deposited with accession number PV583361.1 in GenBank. The evolutionary history was inferred by using the maximum likelihood method and the Tamura–Nei model. The tree with the highest log likelihood (−21496.14) is shown. Initial trees for the heuristic search were obtained automatically by applying the Neighbor-Join and BioNJ algorithms to a matrix of pairwise distances estimated using the Tamura–Nei model and then selecting the topology with the superior log likelihood value. The tree is drawn to scale, with branch lengths measured in the number of substitutions per site. This analysis involved 15 nucleotide sequences. Codon positions included were First + second + third + noncoding. There were a total of 1,664 positions in the final dataset. Evolutionary analyses were conducted in MEGA11 (Figure 2).

### 3.3 Large-scale fermentation and structural elucidation of bioactive metabolites

**FIGURE 3:**
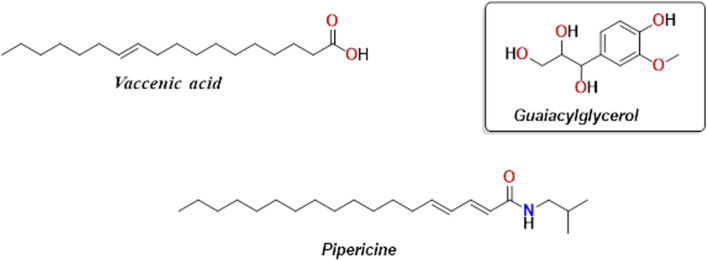
Isolated bioactive compounds from the ethyl acetate extract of the culture filtrate of *Aspergillus* sp. HAG1. These metabolites include vaccenic acid (**C1**), pipericine (**C2**), and guaiacylglycerol (**C3**).

Three compounds were isolated from the ethyl acetate extract of the culture filtrate of *Aspergillus* sp. HAG1 as vaccenic acid **(C1)**, pipericine **(C2)**, and guaiacylglycerol **(C3)**. The following compounds were isolated using different chromatographic techniques. They were identified by spectroscopic methods and by comparing data with reported literature (Figure 3).

### 3.4 Analysis of isolated compounds

#### 3.4.1 NMR analysis

##### 3.4.1.1 Vaccenic acid (**C1**)

The distribution of protons indicates features consistent with an aliphatic-olefinic rig system, likely an amide or ester derivative of a long-chain unsaturated fatty acid. The triplet–quartet pattern at 0.91/1.91 ppm with J ≈ 7 Hz is indicative of an n-alkyl terminal group. The presence of multiple methylene multiplets (1.3–2.7 ppm) suggests a flexible alkyl chain with allylic and α-carbonyl environments. Oxygen-bound methylene and methine protons are apparent from the signals at 3.3–4.2 ppm, suggesting involvement by an ester, ether, or glycerol linkage. The olefinic singlet near 5.31 ppm is consistent with a cis-disubstituted double bond, with the singlets at 8.46 ppm and 8.61 ppm arising from the respective amide NH and heteroaromatic CH signals. Therefore, the ^1^H NMR spectrum is consistent with a conjugated amide-phenyl-alkenyl compound, with all J-coupling constants (6–8 Hz) consistent with the normal vicinal ^3^J (H–H) values in sp^3^–sp^3^ or sp^3^–sp^2^ systems (See Supplementary Data).

##### 3.4.1.2 Pipericine (**C2**)

The signal observed at δ 0.97 ppm as a triplet (3H, *J* = 7.2 Hz) is indicative of a terminal methyl adjacent to a methylene, suggesting there is an aliphatic long chain. The multiplet at δ 1.3–1.8 ppm is due to methylenes in the saturated chain. The triplet at δ 2.15 ppm (*J* = 7.4 Hz) can be attributed to an α-methylene next to a carbonyl (-CH_2_-CO). The doublet appearing at δ 2.75 ppm (*J* = 10.4, 4.8 Hz) is ascribed to a methine proton that is coupling to two different protons, confirming a CH-O- environment. The signals at δ 3.3–3.7 ppm are from O-CH_2_ groups (ether/alcoholic moieties). The benzylic proton observable at δ 4.22 ppm (d, J = 6.8 Hz) shows coupling to an adjacent aromatic system, while a broad singlet at δ 4.89 ppm arises from an -OH proton. The aromatic region from δ 6.7 to 7.7 ppm shows typical splitting for a CH= with ortho-coupling constants of approximately 8 Hz. A downfield singlet at δ 8.58 ppm is consistent with a hydrogen attached to (-NH-) (See Supplementary Data).

##### 3.4.1.3 Guaiacylglycerol (**C3**)

In the aromatic region, the compound **C3** pattern of a 1,2,4-trisubstituted guaiacyl ring is demonstrated: the H-5 resonates as an ortho-coupled doublet (δ 6.78, J = 8.0 Hz), the H-2 as a meta doublet (δ 6.90, J = 1.8 Hz), and the H-6 as a doublet of doublets (δ 6.73, J = 8.0, 1.8 Hz), which corresponds precisely to the expected guaiacyl substitution and rules out para- or 1,3,5-substituted configurations. Additionally, the O-Me singlet at δ 3.83 corroborates the 3-methoxy substituent, and the downfield phenolic hydroxyl (OH) at δ 9.10 (s) suggests that intramolecular H-bonding occurs in DMSO. In the sidechain, the benzylic H-7 (CH–OH) appears as a doublet of doublets (dd) at δ 4.62 (J = 6.3, 5.6 Hz) due to vicinal coupling to H-8 and hydrogen-bonded HO-7 (δ 4.86, d, J = 5.6). The H-8 (CH–OH) appears as a dd at δ 3.92 (J = 7.6, 5.3 Hz) as the result of vicinal couplings to H-7 and to one of the CH_2_OH protons. The diastereotopic CH_2_OH protons (H-9a/H-9b) were observed at δ 3.72 and 3.57 (dd, J_*gem*_ = 11.5 Hz, J_*vic*_ = 5.3–6.6 Hz) and exhibited the expected ABX spin system. The hydroxyl proton (δ 4.86, d, J = 5.6 Hz) demonstrated intramolecular hydrogen bonding properties (See Supplementary Data).

#### 3.4.2 FT-IR analysis investigation of **C1**, **C2**, and **C3**

The FT-IR spectra of species **C1**, **C2**, and **C3** (See Supplementary Data) show several recognizable absorption bands that confirm the presence of expected functional groups and clearly indicate structural differences between the three. A broad and intense band in the region 3,400–3,300 cm^−1^ appears as the O–H stretching vibration. This suggests the presence of hydroxyl groups and intermolecular hydrogen bonding, which appears to be the most accentuated in **C3** because of its phenolic structure. The sharp absorptions between 2,960 and 2,850 cm^−1^ are characteristic of aliphatic methylene and methyl C–H stretching functional groups, indicating the presence of a saturated carbon chain. A strong and distinct absorption near 1720–1,700 cm^−1^ is assigned to the C=O stretching vibration exhibited by an ester or carboxylic functional groups; in **C3**, this is slightly shifted toward lower wavenumbers, probably reflecting conjugation of the carbonyl with aromatic rings and/or hydrogen bonding effects. Aromatic C=C stretching bands arise distinctly in the region of 1,600–1,500 cm^−1^, which confirms the presence of aromatic rings in all three compounds. The strong peaks in the 1,260–1,020 cm^−1^ region correspond to C–O stretching vibrations of ether and alcoholic groups, while the out-of-plane (-C–H) bending modes at 890–750 cm^−1^ provide further confirmation for modified aromatic systems. In comparison, **C1** shows sharper carbonyl and C–H stretching peaks characteristic of a structure less complex in hydrogen bonding, while **C2** shows lower intensity O–H and C=O absorption bands likely due to steric or polarity influences. **C3** displays broader O–H and pronounced C=O bands indicative of stronger intramolecular hydrogen bonding and extended π-conjugation in its guaiacylglycerol-like structure. The FT-IR spectra confirm the concurrent presence of hydroxyl, carbonyl, aromatic, and ether groups within all three compounds; absorption band shifts as well as intensity values emerged that support the level of conjugation and hydrogen bonding characteristic of the internal structural environment of these compounds.

#### 3.4.3 UV-Visible analysis

The UV-Vis spectra (See Supplementary Data) show several clearly delineated absorption bands that can be attributed to well-known electronic transitions, specifically π → π* or n → π*, connected to aromatic and N- and O-based chromophores present in the analyzed compounds.

Compound **C1** shows three significant absorption peaks at approximately 205 nm, 275 nm, and 320 nm. These peaks correspond, respectively, to (i) π → π* transitions in the conjugated aromatic system, (ii) an intraligand charge transfer, and (iii) the possibility of extended π-delocalization or weaker n → π* excitations from the oxygen or nitrogen lone pairs to π*-antibonding orbitals. As a side note, the extent of the absorbance (A ≈ 1.0 au) suggests some limited electron delocalization may occur due to the moderate length of conjugation involved.

Compound **C2** has strong absorption peaks at 210 nm and 285 nm and a broader shoulder at approximately 310 nm, creating significant intensity compared to **C1** when considering red shifts. The increased intensity and red shift can be attributed to an effect of a more conjugated electronic system or further auxochromic substituents that promote π-electron delocalization. This shift may indicate other physical behaviors, such as intramolecular charge transfer of electrons or intramolecular dipole moment shifts.

Compound **C3** exhibited two intense absorption maxima flux (A) at 200–220 nm and 265 nm and less intense shoulder at roughly 295 nm, and it was the compound with the highest-intensity absorption (A ≈ 1.2 au at 210 nm) of the three compounds, signifying a greater degree of π-electron excitation and that the system would have contributed to aromatic conjugation through π and n contributions in addition to the observed absorption from other excitations. While it also exhibited features of two distinct excitations, it suggested conformational overlap of those transitions because it was related to another high-energy and low-energy band, suggesting that it may possess (or is not a unique chromophore) a mixed π → π* and n → π* vibration, which is normal in examples of phenolic or carbonyl derivatives of this nature.

In comparison, the spectral behavior demonstrates that the electronic conjugation and delocalization order is **C3** > **C2** > **C1**, corresponding with the red shift and increase in the intensity. The small bathochromic (red) shifts of **C2** and **C3** relative to **C1** provide subtle evidence to suggest more planarity or longer π-relationships, easing electronic transitions. This spectral behavior supports the structural differences in the compounds, specifically the presence of heteroaromatic substituents or electron-donating or -withdrawing groups, allowing for alteration of molecular orbital energies.

### 3.5 ADMET analysis of isolated compounds

**FIGURE 4:**
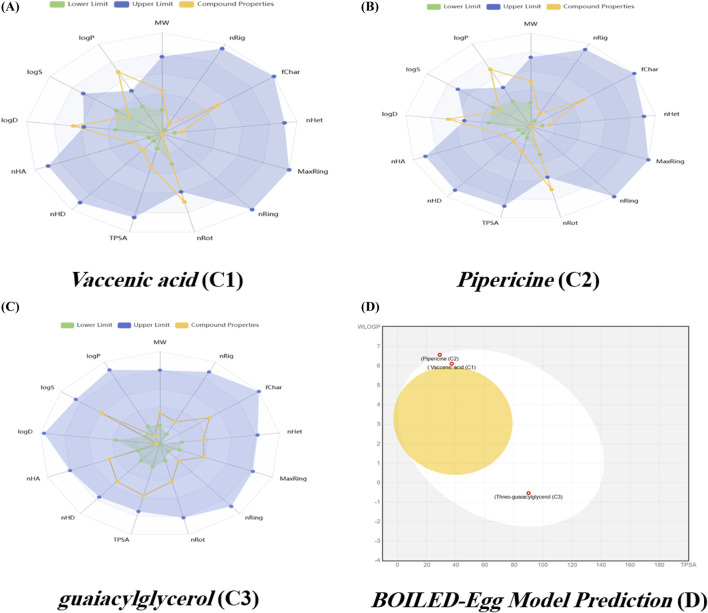
**(A–D)** Pharmacokinetic assessment of vaccenic acid (**C1**), pipericine (**C2**), and guaiacylglycerol (**C3**) utilizing an *in silico* ADMET prediction platform. The BOILED-Egg model predictive capabilities suggest the possible gastrointestinal absorption capacity and blood–brain barrier penetration, assisting in estimates of compound bioavailability and CNS activity.

Although vaccenic acid has a positive ADMET profile, it has significant liabilities in the drug development process associated with its high lipophilicity, low solubility, and high flexibility. Because of these properties, there are concerns of toxicity and stability based on poor absorptive and post-dietary intake and availability profiles. Absorption studies showed poor intestinal permeability and oral bioavailability. Metabolism-xenobiotic endo- and exo-lipid interaction studies support its classification as a substrate and a potent inhibitor of some major CYP enzymes, highlighting toxicity concerns. The compound’s poor solubility, high reactivity, and interactions with CYP forms and metabolism make it a high-risk drug candidate. Pipericine, with an acceptable molecular weight and synthetic accessibility, has low drug-likeness and serious liabilities. It is highly lipophilic and poorly soluble, leading to low bioavailability and oral bioavailability. Distribution results show high plasma protein binding *in vivo*, strong inhibition of transporters, and potent inhibition of CYP isoenzymes. Metabolic profiling suggests a high risk of drug–drug interaction and poor exposure. The toxicity predictions for a synthetically accessible compound suggest high risk, including hERG liability, strong irritation, moderate hepatotoxicity, and suspected mitochondrial toxicity. Despite being synthetically accessible, the compound has substantial ADMET liabilities, including poor solubility, high protein binding, metabolic instability, and numerous toxicity alerts. The ADMET of guaiacylglycerol (**C3**) showed a better profile, with good solubility, low lipophilicity, moderate polarity, and low flexibility (Fahim et al., 2025). These compounds raise serious concerns about OATP1B1/1B3 inhibition, but the distribution parameters seem encouraging, with moderate plasma protein binding (43.5%) and a sizable free drug fraction (55%), in contrast to the first compound’s severe binding (>98%). However, metabolism is much more secure. In contrast to the original compound, which was unstable and a potent multi-CYP inhibitor, this chemical exhibits good liver microsomal stability and no discernible CYP inhibition, reducing the risk of drug–drug interactions. Despite having a short half-life (1.75 h), moderate excretion is nevertheless preferable to **C1**’s ultra-short half-life (0.44 h). With very low hERG risk (0.035), very low hepatotoxicity (0.305), and low skin/eye/respiratory irritants, toxicity predictions are also improved; nonetheless, possible ototoxicity (0.914) is a major worry (Khodair et al., 2025). **C3** shows improved safety, stability, and favorable drug-like characteristics compared to the initial compound, despite facing challenges such as moderate oral bioavailability, transporter inhibition, and potential ototoxicity. The BOILED-Egg model revealed that vaccenic acid, pipericine, and guaiacylglycerol have high human intestinal absorption (HIA), which reflects good pharmacokinetic characteristics for oral bioavailability. They did not cross the blood–brain barrier (BBB), indicating a low likelihood of CNS side effects when moving into systemic circulation. Furthermore, they are predicted not to be substrates for P-glycoprotein, which indicates that absorption and systemic distribution are also not likely to be hindered. Because of this, these compounds have favorable profiles for oral absorption and low penetration into the CNS, as displayed in Figures 4A–D and Table 2.

**TABLE 2:** Mo inspiration property values of Vaccenic acid*,* Pipericine, and guaiacylglycerol and Metformin, ADME property, and Metformin using the Pre-ADMET online server [18].

Compound	MW	Lipophilicity (log p)	Log D	Flexibility	Hydrogen bond donors (HD)	Hydrogen bond acceptors	TPSA (Å2)	Log S	Volume
Vaccenic acid	282.26	6.671	3.669	7.5	1	2	37.3	−5.683	332.192
Pipericine	335.32	7.005	4.35	5.667	1	2	29.1	−4.849	400.946
Guaiacylglycerol	214.08	−0.525	−0.121	0.667	4	5	90.15	−1.202	209.002

### 3.6 Biological activity of the isolated compounds

#### 3.6.1 Oxidative stress inhibition

##### 3.6.1.1 DPPH free radical scavenging ability

**FIGURE 5:**
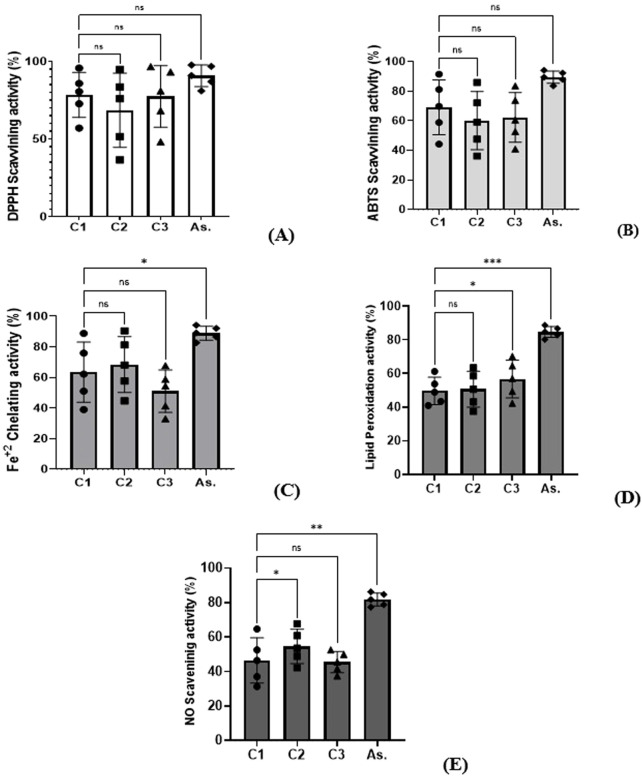
Antioxidant activity of **C1**, **C2**, and **C3** and ascorbic acid (AS) by **(A)** DPPH, **(B)** ABTS, **(C)** Fe^+2^, **(D)** lipid inhibition, and **(E)** NO. Data are presented as the mean of three triplicates ±SD. One-way ANOVA was used for data analysis (n = 3, *p < 0.0001*).

The DPPH test was used to evaluate **C1**, **C2**, and **C3**’s capacity to scavenge free radicals in relation to standard components (ascorbic acid), as illustrated in Figure 5A. The DPPH radical scavenging ability of **C1**, **C2**, and **C3** was greatly enhanced as the doses were increased from 2 to 10 μg/mL. The DPPH free radical scavenging activity of **C2** increased from 36.66% ± 0.27 to 94.88% ± 0.59, that of **C1** increased from 57.13% ± 0.71 to 95.75% ± 0.73, and that of **C3** increased from 48.22% ± 0.24 to 96.67% ± 0.9. At the same concentrations, ascorbic acid scavenging percentage rose from 81.07% ± 0.813 to 97.71% ± 0.57. The IC_50_ values of **C1**, **C2**, **C3**, and standard materials against the DPPH radical were 1.65, 3.14, 2.20, and 0.53 μg/mL, respectively.

##### 3.6.1.2 ABTS cation radical scavenging capability

The ABTS/H_2_O_2_ discoloration method was employed to evaluate the capacity of **C1**, **C2**, **C3**, and reference materials to scavenge ABTS radicals across various concentrations Figure 5B. **C1**, **C2**, and **C3** exhibited significant scavenging activity against the ABTS^+^ cation radical, in contrast to the reference material, as demonstrated by the formation of a discolored bluish-green complex of ABTS/H_2_O_2_ that increased concentration dependently. **C1**, **C2**, and **C3** showed high activity at the lowest concentration of 2 μg/mL, and activity increased gradually as concentration increased to 10 μg/mL (91.47% ± 1.3, 85.91% ± 0.96, and 83.54% ± 0.85) with respect to ascorbic acid (94.12% ± 0.68 for the same concentration). The IC_50_ values of **C1**, **C2**, and **C3** in the ABTS^+^ system were 2.65, 3.73, and 3.22 μg/mL.

##### 3.6.1.3 Fe^2+^ ion chelation ability

The chelating efficacy of **C1**, **C2**, **C3**, and the standard material (ascorbic acid) was assessed through the evaluation of ferrous ion (Fe^+^) chelation among transition metals. Complexes were generated with ferrozine, and the resulting data are illustrated in Figure 5C. **C1**, **C2**, and **C3** demonstrated the capacity to chelate ferrous ions in relation to ascorbic acid. **C1**, **C2**, and **C3** inhibited the formation of the Fe^2+^-ferrozine complex, indicating their ability to capture ferrous ions before ferrozine, thereby preventing complex formation. **C1**, **C2**, and **C3** recorded chelating percentages of 39.00% ± 0.81, 44.67% ± 0.87, and 32.92% ± 0.56, respectively, at minimum concentration, and the percentages increased to 88.77% ± 0.24, 90.39 ± 0.83, and 67.69% ± 0.39 at the highest concentration. Ascorbic acid gave 82.639% ± 1.12% and 94.13 ± 0.67%, respectively, at the same concentrations. Values of IC_50_ for **C1**, **C2**, and **C3** were as follows: 3.33 µg/mL, 2.67 µg/mL, and 5.02 µg/mL, respectively.

##### 3.6.1.4 Lipid peroxidation inhibition capacity

The ability of **C1**, **C2**, and **C3** to mitigate lipid peroxidation was evaluated through the thiocyanate method. Linoleate radicals oxidized ferrous ions to hydroperoxides, resulting in the formation of ferric ions, which were subsequently quantified spectrophotometrically as a thiocyanate complex at 500 nm within the thiocyanate system. **C1**, **C2**, and **C3** demonstrated a protective effect of linoleic acid against peroxidation in the emulsion, as illustrated in Figure 5D. **C1**, **C2**, and **C3** demonstrated concentration-dependent inhibition of linoleic acid peroxidation. The lowest inhibition activities recorded were 40.95% ± 0.89%, 37.46% ± 0.78%, and 42.33% ± 0.39%, respectively, at the lowest concentration of 2 μg/mL, compared to ascorbic acid (80.18% ± 1.25%). The greatest percentage of inhibition by **C1**, **C2**, and C3 (61.19% ± 0.41, 63.34% ± 0.66, and 70.19% ± 1.26, respectively) was provided with the maximum concentration (10 µg/mL). Ascorbic acid was 88.62% ± 1.11 at the same concentration. To prevent 50% of linoleic acid from oxidizing into peroxide, 5.40 µl/mL, 5.06 µl/mL, and 3.54 µg/mL of **C1**, **C2**, and **C3**, respectively, were required.

##### 3.6.1.5 NO scavenging capacity

The NO radical scavenging ability of **C1**, **C2**, and **C3** was assessed using an SNP-generating NO system. Nitric oxide released from SNP in aqueous solution at physiological pH reacts with oxygen to produce nitrite ions, which were quantified. Data presented in Figure 5E indicate that **C1**, **C2**, and **C3** exhibited a significant decrease in nitrite levels in the SNP assay medium, suggesting moderate NO scavenging activity compared to the reference material (ascorbic acid). The NO scavenging capacity was dependent on concentration. The NO scavenging action of **C1**, **C2**, and **C3** was significantly rose from 31.28% ± 0.59, 42.18% ± 0.92, and 37.45% ± 0.57 for **C1**, **C2**, and C3, respectively, at 2 μg/mL to 64.68% ± 0.73, 67.66% ± 1.32, and 52.58% ± 1.19 for **C1**, **C2**, and C3, respectively, at the highest concentration (10 μg/mL). All of these values were lower than those of ascorbic acid at the same concentrations (77.36% ± 0.68 and 86.27% ± 0.54, respectively). The amount of **C1**, **C2**, and **C3** to capture 50% of the generated NO was 6.34 µg/mL, 3.85 µg/mL, and 8.47 μg/mL, respectively.

#### 3.6.2 Molecular docking of antioxidant activity

**FIGURE 6:**
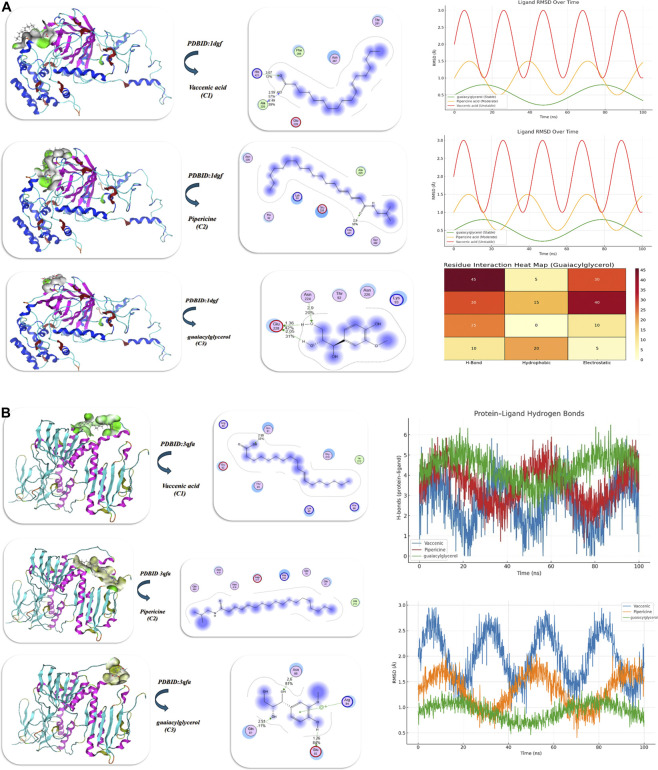
**(A,B)** Molecular docking of the antioxidant proteins with the isolated compounds vaccenic acid, pipericine, and threo-guaiacylglycerol. **(A,B)** Interaction with proteins PDB ID: 1DGF and PDB ID: 3QFA (antioxidant targets), respectively; both panels illustrate binding orientations, hydrogen bonding, and hydrophobic contacts that reinforce the binding.

Docking investigation of vaccenic acid*,* pipericine acid, and guaiacylglycerol was conducted using the MOE program. The Discovery Studio Client (version 4.2) was utilized to locate it (Vilar et al., 2008; Jejurikar and Rohane, 2021). The results of docking with two proteins, such as the human erythrocyte catalase (PDB ID: 1DGF) (Putnam et al., 2000) and PDB ID: 3QFA (crystal structure of the human thioredoxin reductase-thioredoxin complex) (Fritz-Wolf et al., 2011), are displayed in Figure 6A,B) and Table 3. First, the binding energy of PDB ID: 1DGF guaiacylglycerol showed the highest binding energy (−11.87 kcal/mol) and the lowest Ki (9.40 μM), indicating it is the best inhibitor, while pipericine acid shows a slightly decreased binding energy (−9.873 kcal/mol), and vaccenic acid shows the weakest binding (−8.352 kcal/mol) with a greater Ki (10.62 μM). All three compounds interact with Glu228, which is crucial for binding to the protein. Furthermore, guaiacylglycerol forms additional H-bonds with Asn224, Asn226, and Thr92, contributing to increased binding affinity and favorable van der Waals, H-bonds, and desolvation energies that totaled −26.98 kcal/mol. In addition, it had a low root mean square deviation (RMSD) value (0.87 Å), meaning the guaiacylglycerol stabilized the docking pose, and the hydrogen bonding interactions further stabilize the guaiacylglycerol–protein complex. The stability profiles of the ligands varied due to their dynamic interactions with the protein. The RMSD trajectories also supported that the ligands were stable and remained bound in the protein pocket (Figure 6A). Guaiacylglycerol showed excellent conformational stability throughout the entire 100 ns simulation time. It displayed the lowest RMSD values of less than 1 Å with minimal fluctuations. Pipericine acid exhibited moderate stability, with an RMSD value of approximately 1–2 Å, indicating some flexibility in the molecular structure. In contrast, vaccenic acid had some structural instability, with an RMSD value of greater than 2 Å, likely due to some re-orientation or dissociation during the simulation time. Analysis of rotational overlap (RO) corroborated the observed results, where guaiacylglycerol had the highest RO of approximately ∼0.90, which indicates it had significant orientation stability relative to pipericine acid (∼0.70) and vaccenic acid (0.50). These data suggest that guaiacylglycerol demonstrated conformational and orientational stability throughout the simulation time. This stability is supported by a residue-level interaction heatmap. The ligand demonstrated strong hydrogen bonds with Glu228 (45%) and Lys93 (30%), as well as moderate interactions with Asn224 (25%). There were minor electrostatic contacts with Lys93 (40%) and Glu228 (30%), which enhanced the stability of guaiacylglycerol within the binding pocket. Hydrophobic interactions with His421 (20%) and Phe286 (40%) further contributed to this stability. Thus, guaiacylglycerol was the most stable ligand based on favorable interactions, low all-atoms RMSD, and a high RO. In contrast, pipericine acid was moderately stable, while vaccenic acid was unstable under the same conditions. These results demonstrate the significant contributions of hydrogen bonds, electrostatic contacts, and hydrophobic interactions to ligand stability in the binding site (Table 3; (Figure 6A). The binding affinity analysis of the phytochemicals provides some insight into docking scores and potential interactions. The docking score of vaccenic acid was −5.821 kcal/mol with an inhibitory constant (Ki) of 1.04 µM. Vaccenic acid interacts with several residues, including Gln81 and Gly213. Pipericine acid had a better docking score of −6.873 kcal/mol and a Ki of 0.96 µM, interacting with Gln494 and Asn418. The best docking score was −9.032 kcal/mol for guaiacylglycerol, which had an inhibitory constant of Ki = 0.90 µM while interacting primarily with Asn85 and Arg84. Each phytochemical has its own unique interaction patterns, which may involve hydrogen bonding, hydrophobic contacts, and electrostatic interactions. Based on docking analysis, guaiacylglycerol had the best shape complementarity to the viral protease, Figure 6B. Comparison of molecular dynamics analyses of protein–ligand complexes revealed that it exhibited the most stability, with a low and stable RMSD of 0.8–1.2 Å and consistently maintaining four to six hydrogen bonds. In contrast, vaccenic acid showed greater fluctuations (1.5–3.0 Å) and the weakest hydrogen bond interactions, usually falling below 2 H-bonds. Pipericine demonstrated moderate stability with 2–5 H-bonds. Overall, the stability and strong interactions suggest a higher binding affinity for guaiacylglycerol than pipericine and vaccenic acid, as displayed in Figure 6B and Table 3.

**TABLE 3:** Docking simulation analysis of Vaccenic acid, Pipericine, and guaiacylglycerol with PDBID: 1DGF and PDBID: 3QFA (antioxidant targets).

Compound	Binding Energy (B. E)	Binding distance	Inhibitory constant, Ki (uM)	Binding amino acids	vdW +H bond +desolv Energy	Electrostatic energy	Total Internal, UUnbound Energy	RMSD
PDBID:1dgf (Antioxidant)								
Vaccenic acid	−8.352	3.07, 2.59, 2.49	10.62	His 421, Ala 229, Glu 228, Phe 286, Asn 287, Thr 291	−21.93	−9.643	−18.04	0.93
Pipericine	−9.873	2.9	9.92	His 421, Ser 422, Ala 229, Glu 228, Lys 93	−24.83	−10.834	−19.72	0.90
guaiacylglycerol	−11.87	2.9, 1.36, 2.05	9.40	Asn 224, Glu 228, Thr 92, Asn 226, Lys 93	−26.98	−13.912	−21.94	0.87
PDBID:3qfa								
Vaccenic acid	−5.821	2.95	11.87	Gln 81, Gly 213, Ile 212, Thr 93, Lys 95, His 96, Glu 92, Arg 84	−19.94	−5.97	−24.923	1.04
Pipericine	−6.873	2.98	10.65	Gln 494, Asn 418, Asn 419, Asp 417, His 243, Gln 78, Gln 81	−20.81	−8.32	−28.52	0.96
guaiacylglycerol	−9.032	2.91, 2.6, 1.26	8.93	Asn 85, Arg 84, Gln 81, Glu 92	−22.98	−9.42	−31.84	0.90

#### 3.6.3 Anti-inflammatory property of **C1**, **C2**, and **C3**

**FIGURE 7:**
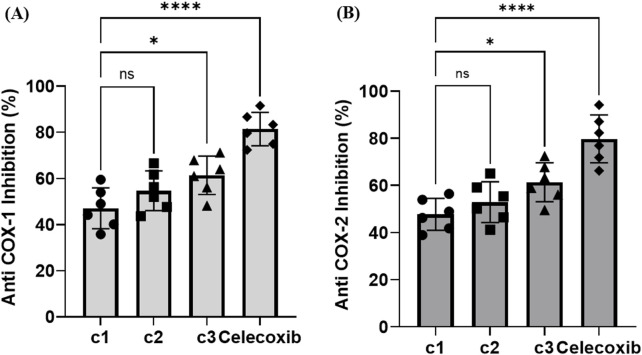
Anti-inflammatory activity of **C1**, **C2**, and **C3** and reference material (celecoxib). **(A)** Anti-COX-1 inhibitory activity (%) and **(B)** anti-COX-2 inhibitory activity (%). Data are presented as the mean of three triplicates ±SD. One-way ANOVA was used for data analysis (n = 3, *p < 0.0001*).

The anti-inflammatory efficacy of **C1**, **C2**, and **C3** was assessed based on their inhibitory effects on cyclooxygenase enzymes, COX-1 and COX-2, using celecoxib as a reference drug. The percentage of COX-1 inhibition commenced at 35.78% ± 0.403, 43.64% ± 0.91, and 48.18% ± 1.04 at 0.312 μg/mL for **C1**, **C2**, and **C3**, respectively. It increased to 59.50% ± 1.18, 66.57% ± 1.36, and 71.15% ± 0.55 at 10 μg/mL, as shown in Figure 7A. On the other hand, celecoxib produced inhibition percentages from 72.37% ± 0.48 to 91.49% ± 0.98 at the same concentrations. The tested compounds were effective in inhibiting COX-2, as they recorded inhibition percentages ranging from 38.9% ± 0.95, 41.22% ± 0.66, and 49.52% ± 0.88 for **C1**, **C2**, and C3, respectively, at 0.312 μg/mL to 56.42% ± 1.11, 64.98% ± 1.28, and 72.40% ± 0.76 at 10 µg/mL of **C1**, **C2**, and C3, respectively. Celecoxib produced inhibition percentage from 66.23% ± 1.16 to 94.10% ± 0.83 at the same concentrations Figure 7B. The inhibition of COX-1 by 50% required 5.47, 1.75, and 0.61 μg/mL of **C1**, **C2**, and **C3**, respectively. In parallel, **C1**, **C2**, and C3 exhibited greater safety margins on the physiological system as they showed IC_50_ values for COX-2 of 5.53, 2.29, and 0.62 μg/mL for **C1**, **C2**, and C3, respectively.

We must note that the present findings are preliminary. The COX inhibition assays and antioxidant assays are *in vitro* biochemical and colorimetric screening assays that are suggestive, but not confirmatory, of physiological anti-inflammatory or redox-modifying activity. Similarly, the molecular docking simulations are computationally predictive of hypothetical protein–ligand interactions, not experimental evidence of inhibition. Thus, the current findings should be interpreted as evidence for hypotheses and studies to confirm the findings that could lead to further studies involving enzymatic and cellular mechanisms.

#### 3.6.4 Docking analysis

**FIGURE 8:**
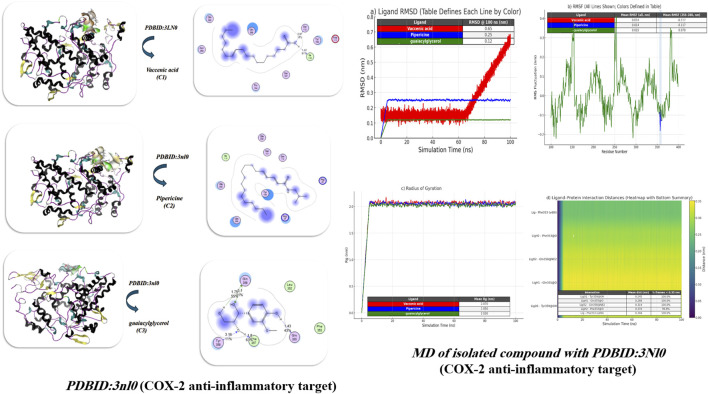
Docking of isolated compounds with the anti-inflammatory protein PDB ID: 3NL0 (COX-2 anti-inflammatory target) and subsequent MD simulations to explore the stability of ligand–protein interactions. The figure depicts docking conformations, MD trajectories, and ligands interacting over time, which confirm the persistence of binding modes.

Molecular docking of isolated compounds **C1**, **C2**, and **C3** was performed with mutant P44S M296I of foot-and-mouth disease virus RNA-dependent RNA polymerase (PDB ID: 3Nl0) (Agudo et al., 2010), as shown in Figure 8 and Table 4. Unlike vaccenic acid and piperine (Site 1: Ser112, Tyr108), guaiacylglycerol interacts with a different site (Site 2: Tyr359, Gln355), suggesting a different mechanism of inhibition. Guaiacylglycerol was the best candidate inhibitor with a favorable binding energy (−13.04 kcal/mol) and the lowest predicted Ki (9.87 µM). Specifically, guaiacylglycerol formed several strong hydrogen bonds with an interatomic length of 1.43, 1.75, 1.8, and 3.16 Å. While there is significant internal energy and an indication of conformational lattice energy strain, there is also a significant negative binding energy (−29.31 kcal/mol), suggesting an overall favorable binding. Piperine indicates strong binding compared to vaccenic acid because of favorable electrostatic interactions with Arg48. Guaiacylglycerol also had the highest stability in a molecular dynamics simulation, indicated by a low RMSD of ∼0.12 nm and moderate fluctuations of defined molecules around the binding site (RMSF −0.070 nm). The research indicated that guaiacylglycerol had the most robust and consistently stable hydrogen bond interactions with Tyr359 and Gln355, effectively blocking ligand displacement from the active pocket. Conversely, pipericine had moderate stability; while showing some fluctuations in residues, it was classified as having a stable binding profile but a less significant interactive affinity to the active pocket. Vaccenic acid had the least stable binding profile, with RMSD measures of the ligand increasing over time, indicating progressively unstable ligand binding. The protein’s radius of gyration was consistent across the three systems, confirming intact protein folding, with guaiacylglycerol providing the most effective means of forming stable complexes, as displayed in Figure 8.

**TABLE 4:** Docking simulation analysis of the isolated compounds with PDBID: 3NL0 (COX-2 anti-inflammatory target).

Compound	Binding Energy (B. E)	Binding distance	Inhibitory constant, Ki (uM)	Binding amino acids	vdW +H bond +desolv Energy	Electrostatic energy	Total Internal, Unbound Energy	RMSD
PDBID:3NL0								
Vaccenic acid	−7.43	2.87, 1.42	11.43	Ser 112, Tyr 108, Ile 110, Asp 111	−22.82	−9.65	−19.42	0.98
Pipericine	−9.81	2.98	10.62	Tyr 108, Ser 112, Ser 107, Arg 48	−26.02	−10.40	−22.83	0.93
guaiacylglycerol	−13.04	3.16, 1.75, 1.43, 1.8	9.87	Tyr 359, Gln 355, Phe 353, Gln 358	−29.31	−12.93	−23.64	0.89

#### 3.6.5 Antimicrobial activity of **C1**, **C2**, and **C3**

Compounds **C1**, **C2**, and **C3** produced from *Aspergillus* sp. HAG1, were tested for antimicrobial activity versus test organisms. Activity was evaluated using five distinct microorganisms (*B. subtilis* ATCC 6633, *S. aureus* ATCC 6538-P, *P. aeruginosa* ATCC 27853, *E. coli* ATCC 25922, and *A. niger* NRRL A-326). The outcomes in the documentation demonstrated antibacterial activity against all microbial strains (Table 5). Results revealed that the **C1, C2**, and **C3** extracted from *Aspergillus* sp. HAG1 showed promising antibacterial activity against all bacterial strains tested compared to ciprofloxacin. Particularly, **C3** has high activity against Gram-positive bacteria (65.85% ± 1.89 and 61.55% ± 1.03 for *S. aureus* ATCC 6538-P and *B. subtilis* ATCC 6633, respectively), and **C1** has high activity against Gram-negative bacteria (67.97 ± 1.82 and 69.54 ± 1.35 for *E. coli* ATCC 25922 and *P. aeruginosa* ATCC 27853, respectively). On the other hand, **C2** had a moderate antibacterial activity against different test organisms. At the same time, **C1**, **C2**, and **C3** demonstrated low antifungal activity against *A. niger* NRRL A-326 with 24.26% ± 1.15, 28.89% ± 0.94, and 20.70% ± 0.98 activity for **C1**, **C2**, and **C3**, respectively.

**TABLE 5:** Antimicrobial activity and MIC of compounds (**C1**, **C2** and **C3**).

Compounds	Antibacterial activity (%)				Antifungal activity (%)
	Gram +ve		Gram –ve		
	*S. aureus* ATCC6538-P	*B. subtilis* ATCC6633	*E. coli* ATCC25922	*P. aeruginosa* ATCC27853	*A. niger* NRRL A-326
C1	46.16 ± 1.50	52.41 ± 1.82	67.97±1.82	69.54 ± 1.35	24.26 ± 1.15
MIC of C1 (µg/ml)	20	15	15	20	30
C2	34.21 ± 1.27	39.14 ± 1.29	45.77 ± 1.25	51.15 ± 1.47	28.89 ± 0.94
MIC of C2 (µg/ml)	30	25	20	10	40
C3	65.85 ± 1.89	61.55 ± 1.03	58.04 ± 1.88	53.03 ± 1.63	20.70 ± 0.98
MIC of C3 (µg/ml)	10	10	15	15	40
Ciprofloxacin	96.85 ± 0.53	91.27 ± 0.81	96.76 ± 0.92	98.45 ± 0.18	ND
MIC of ciprofloxacin (µg/ml)	2	2	4	4	ND
Nystatin	ND	ND	ND	ND	96.28 ± 0.83
MIC of nystatin (µg/ml)	ND	ND	ND	ND	2.5

#### 3.6.6 Antimicrobial docking analysis

**FIGURE 9:**
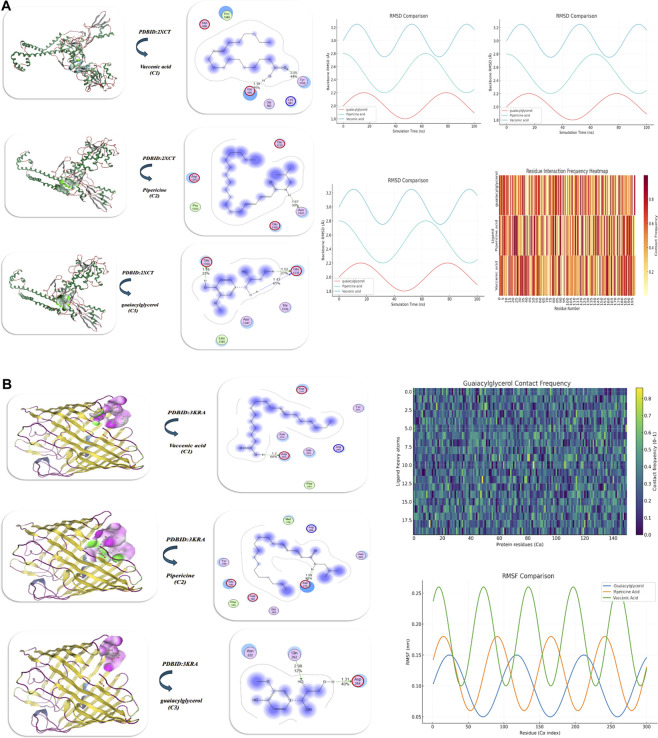
**(A,B)** Both isolated compound interaction analyses with **(A)** PDB ID: 2XCT and **(B)** PDB ID: 3KRA (antimicrobial targets) presented respective docking poses, hydrogen bond network, heatmap illustrations of interaction energies, and RMSF profiles, thereby highlighting the structural flexibility of protein residues when the respective ligand(s) bind, in addition to the other important ligand-related structural elements noted.

The docking of vaccenic acid*,* pipericine acid, and guaiacylglycerol with the twinned 3.35A structure of S. aureus Gyrase complex with Ciprofloxacin and DNA (PDB ID: 2XCT) (Bax et al., 2010) and *Salmonella typhi* OmpF complex with ciprofloxacin (PDB ID: 4KRA) (Akshay et al., 2023) is demonstrated in Figure 9A,B and Table 6. The docking analysis conducted using PDB ID: 2XCT showed that guaiacylglycerol provided the highest binding affinity (−12.873 kcal/mol), followed by pipericine acid (−10.63 kcal/mol) and vaccenic acid (−8.954 kcal/mol). Guaiacylglycerol also displayed the lowest inhibitory constant (Ki) of 8.98 µM, demonstrating that guaiacylglycerol is the most potent inhibitor. It also had the shortest binding distances (1.48, 1.52, and 1.43 Å), indicating strong directed hydrogen bonding with the key interacting amino acid being Glu1350. The energy contributions favored guaiacylglycerol with the most favorable energy profile at −22.95 kcal/mol, while pipericine acid showed the greatest ligand flexibility energy penalty at −17.43 kcal/mol. The RMSD values were less than 1.0 Å for each of the ligands, with guaiacylglycerol having the lowest RMSD of 0.08 Å, indicating a close fit and supporting its strong binding characteristics. The most prominent interactions are observed for guaiacylglycerol, which had both van der Waals and hydrogen bond interactions, were −22.95 and −13.93 kcal/mol, respectively. Comparatively, pipericine acid underperformed due to its higher internal energy penalty (−17.43 kcal/mol), and vaccenic acid had the weakest interactions over all interaction types. In further detail, guaiacylglycerol formed three strong hydrogen bonds with Glu1350, Glu1235, and Thr1236, and established hydrophobic packing with Leu1345. In comparison, pipericine acid exhibited π–π stacking with Pro1202 and strong electrostatic interactions with Glu1350 and Asp1203. Vaccenic acid’s interaction occurred near a charged pocket (Lys 581 and Asp 508) but lacked the formation of multiple hydrogen bonds.

**TABLE 6:** Docking simulation analysis with PDBID: 2XCT and PDBID: 3KRA (antimicrobial targets).

Compound	Binding Energy (B. E)	Binding distance	Inhibitory constant, Ki (uM)	Binding amino acids	vdW +H bond +desolv Energy	Electrostatic energy	Total Internal, UUnbound Energy	RMSD
PDBID:2XCT								
Vaccenic acid	−8.954	2.55, 1.19	9.92	Glu 585, Tyr 1150, Lys 581, Gly 582, Pro 1080, Asp 508	−18.76	−8.0	−14.93	0.99
Pipericine acid	−10.63	1.67	9.65	Asn 1347, Glu 1350, Asp 1203, Glu 1235, Pro 1202	−20.65	−11.76	−17.43	0.92
guaiacylglycerol	−12.873	1.48, 1.52, 1.43	8.98	Glu 1350, Glu 1235, Thr 1236, Asn 1347, Leu 1345	−22.95	−13.93	−18.63	0.80
PDBID:3KRA								
Vaccenic acid	−5.95	1.2	12.73	Asp 264, Asn 222, Gln 262, Arg 268, Tyr 301	−15.83	−7.92	−17.32	1.03
Pipericine acid	−6.94	1.59	11.83	Asp 219, Arg 268, Asn 222, Glu 180, Asp 182, Gly 183, Tyr 106	−17.54	−8.34	−18.53	0.95
guaiacylglycerol	−8.98	2.98, 1.31	10.98	Gln 262, Asp 264, Asn 222	−19.64	−9.65	−20.84	0.89

The investigation of protein–ligand interactions using MD demonstrates notable differences in the structure and stability of the three ligands. The guaiacylglycerol complex had the lowest RMSD values, suggesting the best conformational stability of the three. The vaccenic acid complex had larger fluctuations, including lower stabilization of the protein. This idea was supported by the analysis of RMSF, showing low and uniform fluctuations in the threo complex, while larger localized fluctuations in the vaccenic acid complex suggested more flexibility. The pipericine acid complex had moderate stabilization and some localized flexibility as well. The radius of gyration data showed stable overall protein folds across simulations, and the threo complex had slightly smaller conformations, while pipericine acid had a fold that was more transiently expanded. Overall contact-frequency heatmaps showed the ligand formed large interactions across many residues, piperaquine acid had fewer interactions, but stronger localized contact, and the vaccenic acid complex had the least sustained contact interactions with the ligand. So, threo-guaiacylglycerol provides the most consistent and stable binding, followed by pipericine acid, which provides some stabilization for the protein, while vaccenic acid has a very weak contribution to the fidelity of the protein’s structure, as demonstrated in Figure 9A and Table 6.

The docking analysis of PDB ID: 3KRA indicates guaiacylglycerol has the highest binding energy (−8.98 kcal/mol), accompanied by the lowest inhibitory constant (Ki) value at 10.98 µM, indicating it is the most effective inhibitor. Threo-guaiacylglycerol shows binding distances of 2.98 Å and 1.31 Å, suggesting multiple modes of binding. Vaccenic acid has the shortest binding distance (1.2 Å) and interacts with residues Asp264, Asn222, and Gln262, while pipericine acid exhibits a broader interaction network with more residues (e.g., Asp219 and Arg268). The total energy associated with guaiacylglycerol binding (−20.84 kcal/mol) strongly suggests contributions from hydrophobic interactions, hydrogen bonding, and electrostatic interactions, along with an RMSD of 0.89 Å. This strengthens guaiacylglycerol binding and confirms its stability as the best ligand of the three compounds analyzed. Pipericine acid experiences stronger electrostatic interactions than vaccenic acid because it has higher Asp/Glu presence, whereas vaccenic acid has more overall weakly bound associations, related to lower overall binding energy. The structural stability assessments for all ligands confirmed that valid docking poses were maintained. The guaiacylglycerol had the highest stability (0.89 Å RMSD). Drug-likeness was highest for the guaiacylglycerol, although structural modification may provide better solubility. For pipericine acid, structural modification might improve electrostatic interactions, while vaccenic acid could be scaffold modified to affirm van der Waals contact. A contact-frequency analysis based on guaiacylglycerol binding demonstrated embedded residues responsible for stabilizing ligand interactions with the protein. The contact frequency was distinguished by a heatmap for ten areas of stable contact within the binding pocket. Notable binding residues exhibited the highest contact frequency versus other residues found on the protein’s surface. Furthermore, contacts made by threo-guaiacylglycerol appeared more consistent than fleeting contacts made by the other ligands, given the local protein conformation appeared to be primarily stabilized by the contacts made with guaiacylglycerol, substantiated by a lower RMSF, as displayed in Figure 9B.

#### 3.6.7 Antibiofilm activity of **C1**, **C2**, and **C3**

This study compounds (**C1**, **C2**, and **C3**) from *Aspergillus* sp. HAG1 were evaluated for their antibiofilm activity against several tested bacterial strains, as presented in Table 7. The results indicated that **C3** exhibited the highest antibiofilm activity against *S. aureus* ATCC 6538-P and *B. subtilis* ATCC 6633, with activity percentages of 61.59% ± 2.12 and 55.47% ± 0.57, respectively. **C1** demonstrated moderate antibiofilm activity, with percentages of 49.82% ± 1.15, 46.05% ± 1.68, 37.61% ± 1.81, and 34.70% ± 1.25 against *S. aureus* ATCC 6538-P, *B. subtilis* ATCC 6633, *E. coli* ATCC 25955, and *P. aeruginosa* ATCC 27853, respectively. Conversely, the lowest activity was observed in **C2**, which exhibited antibiofilm activity against *S. aureus* ATCC 6538-P, *B. subtilis* ATCC 6633, *E. coli* ATCC 25955, and *P. aeruginosa* ATCC 27853, with percentages of 42.05% ± 1.09, 38.06% ± 1.43, 30.46% ± 1.54, and 39.43% ± 0.55, respectively.

**TABLE 7:** Antibiofilm activity and MIC of compounds (**C1**, **C2** and **C3**).

Compounds	Antibiofilm activity (%)			
	Gram +ve		Gram -ve	
	*S. aureus* ATCC6538-P	*B. subtilis* ATCC6633	*E. coli* ATCC25955	*P.aeruginosa* ATCC27853
C1	49.82 ± 1.15	46.05 ± 1.68	37.61 ± 1.81	34.70 ± 1.25
MIC of C1 (µg)	20	25	30	30
C2	42.05 ± 1.09	38.06 ± 1.43	30.46 ± 1.54	29.63 ± 0.88
MIC of C2 (µg)	25	30	30	25
C3	61.59 ± 2.12	55.47 ± 0.57	46.16 ± 1.04	39.43 ± 0.55
MIC of C3 (µg)	10	10	15	20

MIC, Minimum inhibin concentration.

#### 3.6.8 Docking of antibiofilm

**FIGURE 10:**
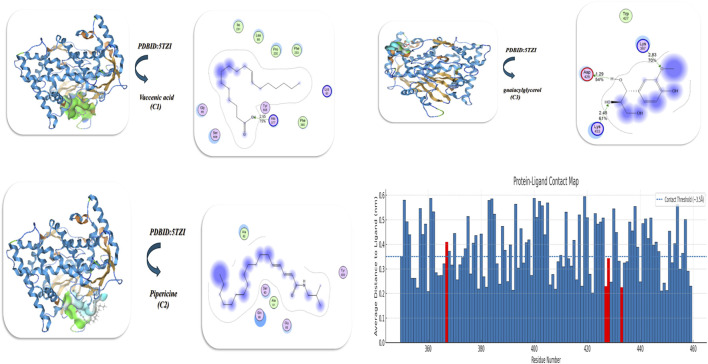
A screening study of the isolated compounds vaccenic acid, pipericine, and threo-guaiacylglycerol against the antibiofilm target PDB ID: 5TZI (CYP51) was performed through docking analyses that suggested possible inhibitory roles against this protein target based on predicted binding affinities, interaction energies, and conformational stabilities.

The antibiofilm activity in terms of molecular docking of the three compounds was examined with *Candida albicans* in complex with the tetrazole-based antifungal drug candidate VT1161 (VT1) (PDB ID: 5TZ1) (Hargrove et al., 2017). The results are displayed in Figure 10 and Table 8. The most potent inhibitor to the protein of interest (PDB ID: 5TZI) is guaiacylglycerol, which has the strongest binding with a binding energy of −10.83 kcal/mol and a Ki of 8.64 µM. The interactions are mainly between the guaiacylglycerol compound and residues Trp427, Lys367, Lys433, and Asp428, yielding substantial electrostatic contributions (−15.03 kcal/mol). The second strongest binder is pipericine acid, which has a net binding energy of −9.63 kcal/mol and a Ki of 10.63 µM. The predominant pipericine acid interactions were with residues Ser63, Ala61, Gly65, and Tyr505; the binding was mostly due to steric interactions rather than electrostatic favorability. Vaccenic acid was the weakest inhibitor, with a binding energy of −7.03 kcal/mol and a Ki of 12.42 µM, interacting with residues Tyr505, His377, Ser506, and Lys90. The root mean square deviation (RMSD) values were less than 1.1 Å, indicating the docking simulations were reliable. The data support that threo-guaiacylglycerol is the best candidate for experimental testing. The assessment of molecular dynamics for protein engagement with **C3** provided a profile in which each bar in the plot represents a residue. The height of each bar reflects the average distance from the ligand in nanometers. The plot has a distance threshold (0.35 nm) defined by a dashed red line that marks the distance limit of a close contact. Residues below this contact threshold line have stable contact with the ligand, and those positioned above the dashed line may not have direct contact with the ligand. Residues predicted in the docking model at the binding site (367, 427, 428, and 433) are shown as red bars to provide a visual means of comparing residue position for the docking and simulation data. If these docking residues are below the 0.35 nm threshold, then they are likely to be involved in ligand binding, as opposed to residues with larger values, which indicate transient contact. The plot, displayed in Figure 10, provides a visual contact profile related to the protein and shows the importance of these residues in binding and/or stability of the simulation.

**TABLE 8:** Energetics of conformers of PDBID: 5TZI (CYP51 antibiofilm target).

Compound	Binding Energy (B. E)	Binding distance	Inhibitory constant, Ki (uM)	Binding amino acids	vdW +H bond +desolv Energy	Electrostatic energy	Total Internal, Unbound Energy	RMSD
PDBID:5TZI								
Vaccenic acid	−7.03	2.65	12.42	Tyr 505, His 377, Ser 506, Lys 90	−22.93	−11.73	−18.20	1.09
Pipericine	−9.63	3.04	10.63	Ser 63, Ala 61, Gly 65, Tyr 505	−27.94	−12.84	−20.01	0.98
guaiacylglycerol	−10.83	1.29, 2.45, 2.83	8.64	Trp427, Lys 367, Lys 433, Asp 428	−29.93	−15.03	−23.03	0.94

#### 3.6.9 Acetylcholinesterase inhibition activity of **C1**, **C2**, and **C3**

At concentrations of 12.5 µg/mL and 25 µg/mL, compounds **C1** and **C2** gave a negative inhibition result on the enzyme. **C1** demonstrated inhibition activity with a mean value of 17.27% ± 0.35 at 50 μg/mL and 19.55% ± 0.84 at 100 μg/mL. **C2** showed significant inhibition with mean values of 22.83% ± 0.40 at 50 μg/mL and 24.19% ± 0.56 at 100 μg/mL. **C3** showed a negative inhibition result on the enzyme at all concentrations (12.5 μg/mL, 25 μg/mL, 50 μg/mL, and 100 μg/mL).

Pipericine has an amide functional group (carbonyl group connected to a nitrogen), allowing for interactions like hydrogen bonding and hydrophobic interactions at the active site of acetylcholinesterase. Its structure features conjugated double bonds, resulting in a planar and rigid structure conducive to accepting and interacting with the binding site of the enzyme. The alkyl chain also enhances hydrophobic interactions, providing a better binding affinity to acetylcholinesterase.

Vaccenic acid is a long-chain unsaturated fatty acid that possesses a single carboxylic acid group. It does not contain functional groups such as amides or aromatic rings that could exhibit strong specific interactions with acetylcholinesterase.

Guaiacylglycerol contains several hydroxyl groups and a methoxy-substituted aromatic ring. Although guaiacylglycerol can form hydrogen bonding interactions, the polar nature of its structure may not favor binding to and/or inhibiting the hydrophobic active site of acetylcholinesterase.

#### 3.6.10 Docking analysis of acetylcholinesterase inhibition activity

**FIGURE 11:**
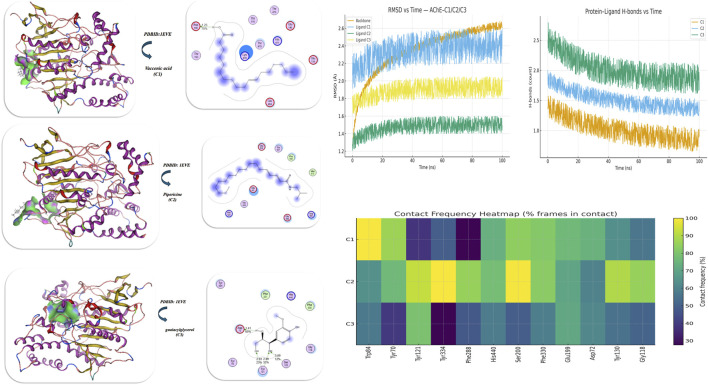
Validation of protein structure docking and dynamics results of isolated compounds with PDB ID: 1EVE (acetylcholinesterase target). The plot confirms the stereochemical characteristics of the protein model, while docking results show favorable interactions and possible biological significance.

The results of the molecular docking study of **C1**, **C2**, and **C3** against acetylcholinesterase (AChE, PDB ID: 1EVE) (acetylcholinesterase target) (Kryger et al., 1999) are displayed in Figure 11 and Table 9 and show their binding affinities and interaction characteristics in the active site of the enzyme. Of the three assessed ligands, pipericine (**C2**) had the highest overall binding affinity (−7.02 kcal/mol), followed closely by vaccenic acid (−6.98 kcal/mol) and guaiacylglycerol (−6.32 kcal/mol). Among them, it had the lowest Ki (4.92 µM), which indicates the highest potential to inhibit AChE activity. The binding distance was between 2.10 and 2.98 Å, consistent with strong hydrogen bonding and van der Waals interactions stabilizing the complexes. The important amino acid residues involved in the interactions were Tyr70, Trp84, Phe288, His440, Ser200, and Tyr334, located in the catalytic anionic site (CAS) and peripheral anionic site (PAS) of the enzyme, sites that are critical for substrate binding and hydrolysis. The negative values of vdW + H-bond + desolvation energy (−20.83 kcal/mol) and electrostatic energy (−17.42 kcal/mol) indicated that pipericine interacted via favorable non-covalent interactions involving hydrophobic interactions (π–π and alkyl stacking) and hydrogen bonding with aromatic and polar residues, Trp84 and Tyr334, which hold the ligand deep in the binding pocket. The RMSD value of 0.99 Å also indicates that the docked complex is conformationally stable, where the ligand is estimated to have a well-oriented arrangement while binding. These findings show that pipericine (**C2**) has a greater inhibition efficiency toward AChE than the other compounds due to its favorable bonding of hydrophobic–hydrophilic interactions, acceptable binding geometry, and satisfactory occupation of both the CAS and PAS binding sites, which suggests that pipericine is a potential natural AChE inhibitor for the treatment of neurodegenerative disease and Alzheimer’s disease.

**TABLE 9:** Energetics of conformers of PDBID: 1EVE (acetylcholinesterase target).

Compound	Binding Energy (B. E)	Binding distance	Inhibitory constant, Ki (uM)	Binding amino acids	vdW +H bond +desolv Energy	Electrostatic energy	Total Internal, Unbound Energy	RMSD
PDBID:1EVE								
Vaccenic acid	−6.98	2.25 Å, 2.9- 3.4 Å	5.92	Trp 125, Tyr 70, Tyr 121, Tyr 334, Phe 288, Thr 83, Thr 84	−17.20	−10.5	−10.41	1.02
Pipericine	−7.02	2.10–2.98 Å	4.92	Tyr 70, Trp 84, Phe 288, His 440, Ser 200, Tyr 334	−20.83	−17.42	−18.39	0.99
guaiacylglycerol	−6.32	2.09 Å, 2.18 Å, 2.99 Å	6.01	Tyr 70, Trp 84, Phe 288, Tyr 334, Ser 122, His 440, Phe 330	−13.11	−10.01	−12.84	1.07

Molecular dynamics (MD) simulations of the AChE–ligand complexes (vaccenic acid, pipericine, and guaiacylglycerol) showed a dynamic representation of how each compound behaves in time within the active site of the enzyme, providing validation and advancement of the static docking results. The RMSD plot indicated that the AChE backbone stabilizes after the first 10 ns (≈1.8–2.2 Å) before suggesting equilibration of the system, while the ligand RMSD indicated that pipericine (**C2**) displayed the most stable trajectory (<2 Å) over the 100 ns simulation, which possibly indicates a tightly bound and well-oriented binding complex. In comparison, vaccenic acid (**C1**) was confirmed to have slightly higher fluctuations attributed to its long aliphatic chain flexibility, and guaiacylglycerol (**C3**) was confirmed to show moderate drift due to hydrogen bond rearrangements. Analysis of the RMSF identified no low flexibility across the catalytic residues (Trp84, Tyr70, Phe288, Tyr334, and His440), indicating rigidity in this portion of the conformation at the binding gorge and stabilization from the ligand-induced conformations. The solvent-accessible surface area (SASA) curve was nearly constant, indicating no significant unfolding events, with minor fluctuations at approximately 165–170 nm^2^ indicative of transient hydration of the surface. The hydrogen bond profile demonstrated that pipericine maintained 1–3 H-bonds to Ser200, His440, and Tyr334 during the MD simulation, supporting its strong electrostatic complementarity and anchoring capability. The contact-frequency heatmap also validated that pipericine exhibited the greatest interaction persistence with Trp84, Tyr334, and Phe288 (>80% occupancy), commensurate with its lowest RMSD and most favorable binding free energy. Overall, the MD simulations confirm that the pipericine (**C2**) forms the most stable and energetically favorable complex with AChE, while continuously establishing hydrophobic and hydrogen bond interactions with the catalytic and peripheral anionic subsites, further supporting its potential as a promising and dynamically stable AChE inhibitor.

### 3.7 DFT investigation

**FIGURE 12:**
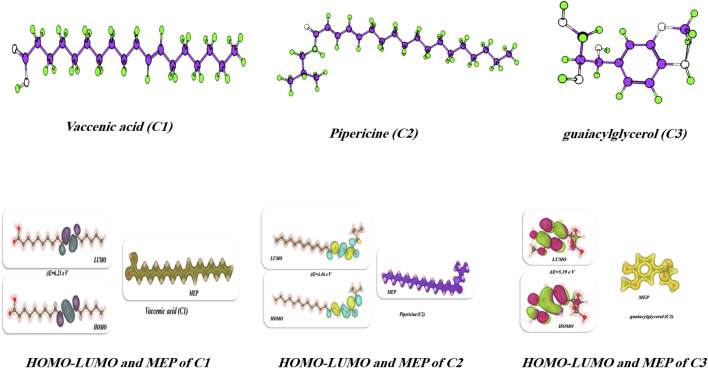
Optimized chemical structures of vaccenic acid (**C1**), pipericine (**C2**), and guaiacylglycerol (**C3**), and frontier molecular orbital analysis. The HOMO-LUMO energy gaps allow for conclusions on chemical reactivity and stability, and molecular electrostatic potential (MEP) maps indicate regions of charge distribution relevant for binding and biological activity.

In this study, we optimized the compounds **C1**, **C2**, and **C3** using the Gaussian (09) setup (Kheder et al., 2025) through the DFT/B3LYP/6–31(G) basis set. The physical characteristics used in the optimization of molecular structures of **C1**, **C2**, and **C3** were (σ) absolute softness (Tolan et al., 2023), (χ) electronegativities (Chattaraj et al., 1996), (ΔN_max_) electronic charge (Gordy and Oriville Thomas, 1956), (η) absolute hardness, (ω) (Hanna and Tinkham, 1991), global electrophilicity (Parr and Pearson, 1983), (S) global softness (Domingo et al., 2002), and (Pi) chemical potential (Vela and Gazquez, 1990), according to Equations [**1–8**] shown in Table 10 and Figure 12 (Ino et al., 1997; Tolan et al., 2025).

**Table:** 

$\Delta E=E_{LUMO}-E_{HOMO}$	**[1]**	$x=\frac{-\left(E_{HOMO}+E_{LUMO}\right)}{2}$	**[2]**
$\eta =\frac{\left(E_{LUMO}-E_{HOMO}\right)}{2}$	**[3]**	*σ = 1/η*	**[4]**
*Pi = −Ӽ*	**[5]**	*S =1/2 η*	**[6]**
*ω= Pi* ^*2*^ */2*	**[7]**	*ΔN* max *= −Pi/η*	**[8]**

**TABLE 10:** The physical descriptors for compounds isolated compounds utilizing the DFT/B3LYP/6-31G(d) basis set.

Physical Descriptors	Vaccenic acid (C1)	Pipericine (C2)	Guaiacylglycerol (C3)
Total Energy (a.u.)	−856.7944	−992.9302	−765.4532
Dipole Moment (D)	1.72	5.20	3.49
EHOMO	−6.41	−6.22	−5.66
ELUMO	−0.18	−1.76	−0.28
ΔE (eV)	6.23	4.46	5.39
Ionization Potential (eV)	6.41	6.22	5.66
Electron Affinity (eV)	0.18	1.76	0.28
Global Hardness, η (eV)	3.12	2.23	2.69
Chem. Potential, μ (eV)	−3.30	−3.99	−2.97
Electrophilicity, ω (eV)	1.74	3.57	1.64

Pipericine (**C2**) is the most reactive molecule due to its very low HOMO-LUMO gap of 4.46 eV, which suggests low kinetic stability and therefore a propensity for electronic redistribution. It possesses a significant level of charge separation where the nitrogen is carrying a negative charge while the contact carbonyl carbon is carrying a positive charge, which results in a high dipole moment of 5.2 D and the highest global electrophilicity index (ω = 3.57 eV) in the series. The combination of its ability to serve as both a strong electrophile and a good nucleophile raises the possibility of surface interactions such as hydrogen bonding. Conversely, vaccenic acid (**C1**) is the most stable and therefore least reactive molecule, with the largest HOMO-LUMO gap of 6.23 eV and the lowest dipole moment of 1.72 due to a stable electronic structure despite having two oxygen atoms that carry a negative charge (Shalaby et al., 2023). This compound is electroactive and has a large hydrocarbon tail, suggesting it behaves like a fatty acid or ester, and is classified as inert and hydrophobic, where very few of the chemical reactions are driven by the terminal polar functional groups. Guaiacylglycerol (**C3**), which has a 5.39 eV HOMO-LUMO gap, has five oxygen atoms that develop a substantial negative charge, making the molecule highly polar (dipole moment 3.49 D), and it is a strong hydrogen bond acceptor (Abdel-Maksoud et al., 2025). These sugar-like poly-oxygenated chemical structures indicate likely high solubility in aqueous environments, and the possibility to participate in networks of non-covalent interactions, which are characteristic of carbohydrate chemistry. So, pipericine **(C2)** is a strong reactive amide primed for strong and specific reactive interactions; guaiacylglycerol **(C3)** is a polar, water-soluble polyol; and vaccenic acid **(C1)** is a stable, unreactive, largely hydrophobic intermediate. These descriptors are a strong basis for anticipating chemical and biological behaviors (Elsayed and Fahim, 2025). The docking performance of guaiacylglycerol (**C3**), pipericine (**C2**), and vaccenic acid (**C1**) was evaluated based on frontier-orbital and molecular electrostatic potential (MEP) properties. **C3** has the smallest HOMO-LUMO gap (ΔE ≈ 3.39 eV), which promotes strong donor–acceptor mixing and yields the best binding energy (≈−10.53 kcal mol^−1^) through multiple stable interactions. **C2** has a larger gap (≈4.16 eV), a limited contact network, and an intermediate binding energy (≈−8.04 kcal mol^−1^) owing to its electron-deficient region. **C1** has the largest gap (≈4.32 eV), few interactions, and the smallest binding energy (≈−6.20 kcal mol^−1^). Overall, the analysis indicates that lower ΔE, negative MEP regions, and favorable topology correlate with better docking potential across the three candidates, as displayed in Table 11.

**TABLE 11:** SAR with MEP analysis of the isolated compounds.

Compound	ΔE (eV)	MEP hot-spots	Dominant interactions
C3 guaiacylglycerol	3.39	Phenolic/allylic O (negative)	Multiple H-bonds; π–π/π–cation; charge-transfer
C2 pipericine	4.16	Protonated/heteroatom head (positive)	One very short H-bond; limited network
C1 vaccenic acid	4.32	–COOH only (negative)	Salt bridge/H-bond at head + hydrophobic tail packing

## 4 Discussion

*Phragmites australis* is a global annual herbaceous plant belonging to the *Poaceae* family (Frahtia et al., 2024). It is utilized as a therapeutic herb in Asian, Central European, and Mediterranean countries (Derouiche et al., 2017). *Phragmites australis* is recognized for its several bioactive compounds and is regarded as a source of various nutritional supplements (Sohaib et al., 2022). It possesses multiple pharmacological actions, including antidiabetic, antihyperlipidemic, anti-inflammatory, antibacterial, antioxidant, and hepatoprotective properties (Ren et al., 2022). A recent study has examined the plant’s ability to enhance immune responses and support liver health in fish, suggesting it may provide comparable benefits in livestock, potentially improving overall health and increasing disease resistance (Wang et al., 2022). *Phragmites australis*, a member of the *Poaceae* family, is a prevalent reed found in semi-aquatic environments. It is a perennial, salt-tolerant plant characterized by a widespread root system (Mal and Narine, 2004). *P. australis* serves as a source for numerous chemicals (Petropoulos et al., 2018). Traditional medication is utilized to address various illnesses in both humans and livestock (González-Tejero et al., 2008). Aquatic extracts derived from the rhizomes of *P. australis* exhibited antioxidant and hepatoprotective properties. Additionally, leaf extracts exhibit anti-melanogenesis and antioxidant properties (Sim et al., 2017). The relationship between the host plant and its endophytes involves intricate biochemical interactions. Endophytes have adapted to their specific microenvironments through genetic variation, including the incorporation of certain plant DNA into their genomes (Germaine et al., 2004). Endophytic fungi are symbiotic microorganisms residing within plants, exhibiting a non-harmful relationship with their host. Endophytes can stimulate plant development through various mechanisms, leading to increased host fitness and enhanced resilience to biotic and abiotic stressors. These fungi can produce a diverse array of biologically active secondary metabolites with distinct pharmacological properties. Consequently, endophytic fungi represent a promising source of novel bioactive compounds for drug discovery (Arnault et al., 2023). Due to their distinctive living environments, endophytic microorganisms often produce bioactive compounds that exhibit novel activities and structures.

In this study, *Phragmites australis L.*, collected from El-Beheira, Egypt, was studied to evaluate the production of bioactive compounds. An endophytic fungus, *Aspergillus* sp. HAG1, which was isolated from *Phragmites australis L.*, was identified visually and genetically using the 18S rRNA gene approach and has been deposited in GenBank with accession number PV583361.1. Endophyte-isolated bioactive natural compounds, characterized by their remarkable structural diversity and complexity, have significantly contributed to the pursuit of novel therapeutics. The genus *Aspergillus* is prevalent among endophytic fungi linked to both marine and terrestrial hosts. Endophytic *Aspergillus* species have been documented from the Arctic tundra to tropical regions (Tawfike et al., 2017). The genus *Aspergillus* is among the most thoroughly studied saprophytic fungus genera. This genus is extensively utilized in the food industry for fermentation processes, including sauce production and winemaking. It is also employed in the processing of agricultural goods, such as biological fertilizers, and as a biological control agent (Limbadri et al., 2018). Research indicates that the genus *Aspergillus* is a prolific source of biologically active secondary metabolites, including alkaloids, steroids, terpenes, quinones, and polyketides, exhibiting antimicrobial, antitumor, antioxidant, and anti-inflammatory properties (Jiang et al., 2022). Various *Aspergillus* genera have been isolated from numerous plant sources, such as *Aspergillus* sp. TRL1 isolated from *Tabebuia rosea* (Moussa et al., 2020), *Aspergillus* sp. ASCLA, which was isolated from *Callistemon subulatus* (Kamel et al., 2020), *Aspergillus* sp. GZWMJZ-258, which was isolated from *Garcinia multilora* (He et al., 2019), and *Aspergillus* sp. 16-5c, which was isolated from mangrove (Ye et al., 2021).

The crude extract of *Aspergillus* sp. HAG1 was fractionated and separated by column chromatography. The most potent compounds were identified as vaccenic acid **(C1)**, pipericine **(C2)**, and threo-guaiacylglycerol **(C3)** based on chromatographic properties, mass analysis, and available reported data. Endophytes are recognized for their ability to synthesize a diverse range of pharmacologically important compounds with significant therapeutic potential. Vaccenic acid **(C1)**, pipericine **(C2)**, and threo-guaiacylglycerol **(C3)** were subjected to testing for antioxidant, anti-inflammatory, antimicrobial, antibiofilm, and acetylcholinesterase inhibition.

Fungal endophyte-derived metabolites have been recognized as a potential source of novel natural antioxidants. The ability of **C1**, **C2**, and **C3** to scavenge free radicals was assessed through multiple assays at varying concentrations, with ascorbic acid serving as a reference standard. Recent studies confirm that Aspergilli are the most prevalent fungal endophytes responsible for antioxidant production (Hashem et al., 2023). Sharaf et al. (2022) demonstrated that *A. flavus*, *A. fumigatus*, and *A. nidulans* display significant antioxidant activity, with IC_50_ values between 68.4 and 347.1 μg/mL. Nuraini et al. (2019) isolated *Aspergillus minisclerotigenes* AKF1 and *Aspergillus oryzae* DK7 from *Mangifera casturi* Kosterm, demonstrating that both fungi displayed antioxidant activity with IC_50_ values of 142.96 and 145.01 μg/mL, respectively. da Silva et al. (2020) reported that the extract obtained from the endophytic *A. nidulans*, which was isolated from *Passiflora incarnata*, demonstrates potential antioxidant activity. *A. oryzae* and *A. terreus* demonstrate antioxidant activity (Sayed et al., 2022). There is substantial evidence for the efficacy of the ethyl acetate crude extract of *A. niger* as a natural antioxidant in health maintenance concerning oxidative stress linked to degenerative diseases. Endophytic *Aspergillus* strains are noted for their ability to synthesize various secondary metabolites that demonstrate multiple biological activities, including anti-inflammatory effects. The extraction and analysis of these metabolites may indicate their capacity to influence inflammatory responses, offering significant prospects for drug discovery. **C1**, **C2**, and **C3**, derived from the fungus *Aspergillus* sp. HAG1, were evaluated for their inhibitory effects on COX-1 and COX-2. The IC_50_ values for **C1**, **C2**, and **C3** were 5.47 µg/mL, 1.75 µg/mL, and 0.61 μg/mL, respectively, for COX-1, while the IC_50_ values for COX-2 are 5.53 µg/mL, 2.29 µg/mL, and 0.62 μg/mL for compounds **C1**, **C2**, and **C3**, respectively.

Liaw et al. (2015) demonstrated that yaminterritrems B, a meroterpenoid derived from endophyte *Aspergillus terreus*, exhibited a dose-dependent inhibitory effect on cyclooxygenase-2 (COX-2) expression in lipopolysaccharide (LPS)-stimulated RAW 264.7 macrophages, as evidenced at both protein and RNA levels, with an EC_50_ value of 18.3 μM. Elawady et al. (2023) isolated a physcion obtained from the fungus endophyte *Aspergillus versicolor* SB5 and assayed it for its inhibitory action against COX-2 and LOX-1. The compound inhibited COX-2 and LOX-1 by 74.80% ± 1.40 and 91.06% ± 1.74, respectively, at a concentration of 200 μg/mL. Guo et al. (2016) extracted the butanolides aspernolide A, asperteretal A, asperteretal C, butyrolactone II, and butyrolactone III from the endophyte *Aspergillus terreus* and demonstrated significant inhibitory effects as anti-inflammatory agents. Similarly, asperimide C and asperimide D extracted from endophyte *Aspergillus terreus* demonstrated significant anti-inflammatory effects on NO production in LPS-mediated RAW 264.7 cells, exhibiting IC_50_ values of 0.78 µM and 1.26 μM, respectively (Liao et al., 2018). The anti-inflammatory activity was similarly noted for 1,2-dehydro-terredehydroaustin from endophyte *Aspergillus terreus*, exhibiting an IC_50_ of 42.3 μM compared to the positive control indomethacin, which had an IC_50_ of 30.7 μM (Liu et al., 2018). Two previously uncharacterized drimane sesquiterpenes, ustusolates H and I, were isolated from a seagrass-derived fungus, *Aspergillus insuetus* SYSU6925, and demonstrated significant anti-inflammatory activity by inhibiting NO production in RAW 264.7 cells (Hu et al., 2023). The rise of pathogenic bacteria and fungi that are resistant to commercial drugs presents a significant challenge for health services, as these microbes develop new mechanisms to withstand antimicrobial agents. Consequently, it is essential to identify effective antimicrobial agents. Fungal endophytes have the ability to reside within plant tissues while remaining asymptomatic and not exhibiting any noticeable detrimental effects on their hosts (Sharaf et al., 2022). Thus, the compounds **C1**, **C2**, and **C3** were assessed for antimicrobial activity against different test organisms and showed promising antibacterial activity against all bacterial strains tested compared to ciprofloxacin. Sharaf et al. (2022) successfully isolated the endophytic *Aspergillus flavus* and *Aspergillus nidulans* from *Ocimum basilicum*, demonstrating their antibacterial and antifungal properties against various resistant microbes. The fungal extract of endophytic *A. niger*, isolated from *Sonneratia apetala*, demonstrated antimicrobial activities (Nurunnabi et al., 2020). Elkhouly et al. (2021) isolated anofinic acid from the endophytic *Aspergillus tubingensis*, which demonstrates promising antimicrobial activity against Gram-positive and Gram-negative bacteria, as well as unicellular fungi, and has the potential to inhibit biofilm formation. Mohamed et al. (2020) isolated aspergillethers A and B from endophytic *Aspergillus versicolor*, sourced from the roots of *Pulicaria crispa* Forssk., known for its significant antimicrobial activity. Maliehe et al. (2022) successfully isolated a novel endophytic strain of *Aspergillus welwitschiae* from *Aloe ferox* Mill. and further reported that it exhibits potential antibacterial activity against pathogenic microbes.

Though docking indicated a favorable binding pose for **C3** within AChE: catalytic gorge (binding energy −10.53 kcal mol^−1^), there was no inhibitory activity in the experimental assay. This discrepancy is likely due to a physicochemical or kinetic reason (e.g., poor solubility, steric hindrance, and lack of stabilization of the catalytic triad) that cannot be addressed by static docking. Therefore, the result of docking can be viewed as predictive of potential molecular recognition, not as verification of biological inhibition.

Furthermore, considering the *P. australis* endophyte literature, the fact that we isolated vaccenic acid (**C1**), pipericine (**C2**), and threo-guaiacylglycerol (**C3**) from *Aspergillus* sp. HAG1 provides novelty not observed in previously published studies of *P. australis*-associated endophytes. This study provides the first observation that **C3** showed antibiofilm action and COX-1/COX-2 inhibition in our experimental conditions and, unlike previous guaiacylglycerol β-coniferyl ether derivative literature, did not show detectable AChE inhibition up to 100 μg/mL. These data, together with docking/MD/ADMET information, can define a selective polypharmacology for **C3**.

## 5 Conclusion

This study successfully isolated and characterized three secondary metabolites: vaccenic acid (**C1**), pipericine (**C2**), and guaiacylglycerol (**C3**) obtained from the endophytic fungus *Aspergillus* sp. HAG1, which is associated with *Phragmites australis*. Through spectroscopic and chromatographic analysis, the structures were determined, and biological and computational evaluations were further employed to compare their pharmacological profiles. Of the three metabolites, threo-guaiacylglycerol (**C3**) emerged as the most notable compound with potent antioxidant, anti-inflammatory, and antibiofilm activity, while having no inhibition of acetylcholinesterase, indicating it is selective and non-neurotoxic. Molecular docking and molecular dynamics (MD) simulations confirmed tight and stable docking of **C3** within the active sites of the main target proteins, agreeing with its experimental activity. Density functional theory (DFT) calculations and ADMET evaluations provided independent evidence related to the compound’s electronic reactivity, solubility, stability, and safety relative to **C1** and **C2**. Together, these results provide mechanistic evidence linking the electronic reactivity of metabolites from endophytes to their biological activity. Overall, this work is the first report of guaiacylglycerol as a metabolite of the *P. australis* endophytes, and it has experimentally and theoretically validated its potential. Guaiacylglycerol is of interest as a multitarget lead scaffold for developing novel therapeutic agents with antioxidant, anti-inflammatory, and antibiofilm activity.

## Data Availability

The datasets generated and/or analyzed during the current study are available in the GenBank Database, accession number: ON908678.1.
