# Supplementary material for: Isolation, characterization, and biological evaluation of endophytic fungi from Phragmites australis: experimental and computational insights
Source: Front Mol Biosci. 2026 Jan 9;12:1713876. doi: 10.3389/fmolb.2025.1713876 (PMC12827186; doi:10.3389/fmolb.2025.1713876)
Supplement: Supplementary file 1 [file Supplementaryfile1.docx]

***Supplementary materials:***

**Isolation, Characterization, and Biological Evaluation of Endophytic Fungi from *Phragmites australis*: Experimental and Computational Insights**

**Dina Mahfouz Eskander^1^, Mohamed E. El Awady^2^, Mohamed Ali^3^, Asmaa M. Fahim^4^, Ahmed A. Hamed^5*^, Basel Sitohy ^6,7*^**

**^1^** Chemistry of Natural Compounds Department, National Research Centre, Dokki, 12622, Cairo, Egypt.

**^2^** Microbial Biotechnology Department, National Research Centre, 12622 Dokki, Cairo, Egypt.

**^3^** Biochemistry department, faculty of science, Zagazig university 44519, Zagazig, Egypt.

**^4^** Department of Green Chemistry, National Research Centre, Dokki, P.O. Box 12622, Cairo, Egypt.

**^5^** Microbial Chemistry Department, National Research Centre, 12622 Dokki, Cairo, Egypt.

**^6^** Department of Clinical Microbiology, Infection, and Immunology, Umeå University, SE-90185 Umeå, Sweden.

**^7^** Department of Diagnostics and Intervention, Oncology, Umeå University, SE-90185 Umeå, Sweden.

***Correspondence:** [ahmedshalbio@gmail.com](mailto:ahmedshalbio@gmail.com) and basel.sitohy@umu.se.

***1HNMR analysis:***


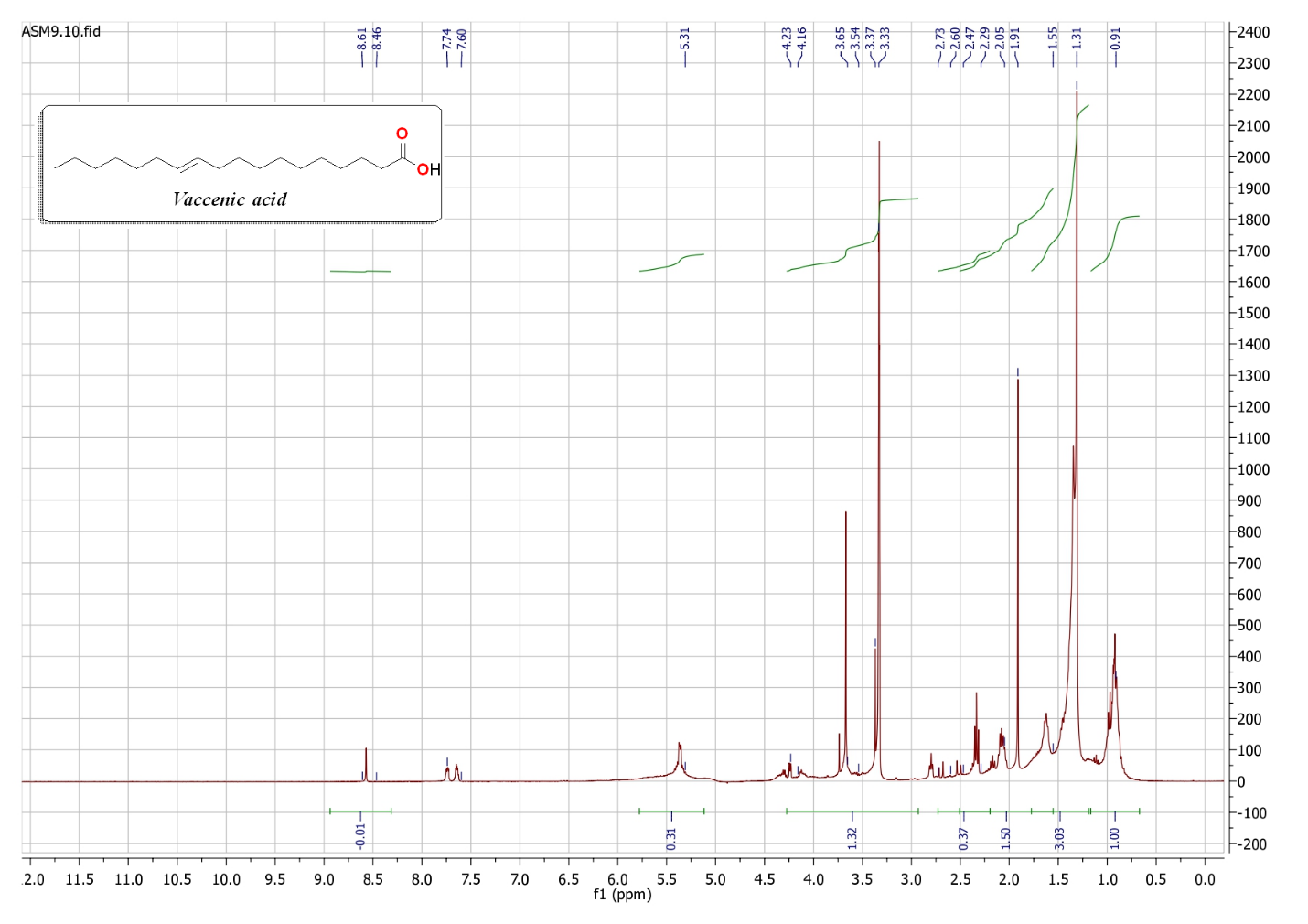


Table.S1: Compact coupling of Vaccenic acid:

| **Proton Type** | **δ (ppm)** | **Multiplicity** | **Integration** | **Coupled Partners** |
| --- | --- | --- | --- | --- |
| –CH₃ | 0.91 | t | 3 H | –CH₂– |
| –CH₂– (β–γ) | 1.31–1.55 | m | 8 H | –CH₂–, –CH₃ |
| –CH₂– next to C=O | 1.91 (q) | 2 H | –CH₂–CH₃ | 7.2 |
| –CH₂– α to C=C | 2.05–2.47 (m) | 4 H | –CH=CH– | 6–8 |
| –CH₂– allylic | 2.60–2.73 (m) | 2 H | –CH=CH– | 7.0 |
| –CH₂–O– | 3.33–3.65 (m) | 2 H | –CH₂–CH–O– | 6.4 |
| CH–O–C | 4.16–4.23 (m) | 1 H | –CH₂–O– | 6.0 |
| =CH– | 5.31 (br s) | 1 H | none | – |
| –NH– | 8.46 (s) | 1 H | – | – |


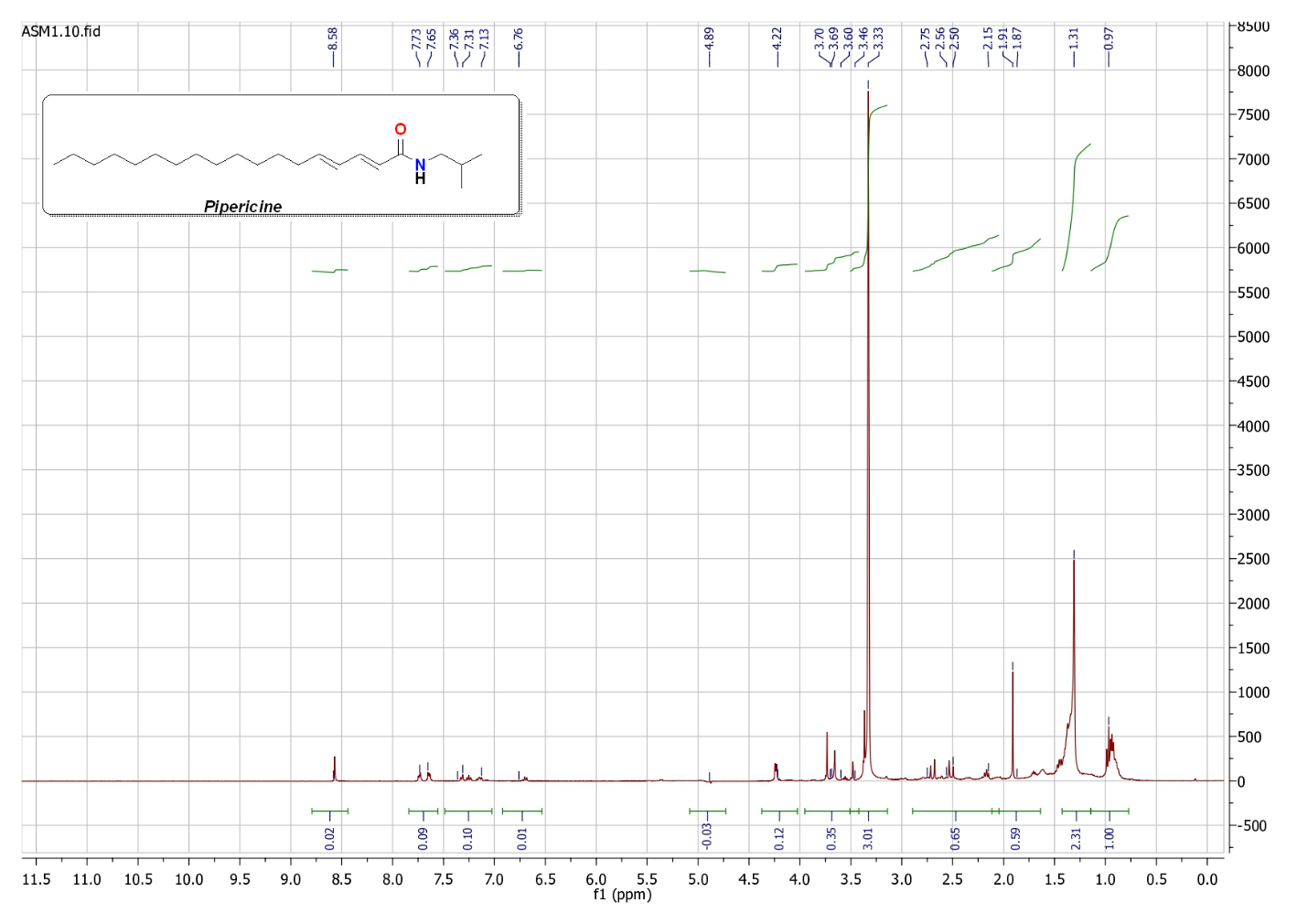


Table.S2: Compact coupling of Pipericine:

| **Signal (δ, ppm)** | **Multiplicity** | **J (Hz)** | **Type of Coupling** |
| --- | --- | --- | --- |
| 0.97 | t | 7.2 | ³J_H–H (vicinal) |
| 2.15 | t | 7.4 | ³J_H–H (vicinal) |
| 2.75 | dd | 10.4, 4.8 | ²J_gem & ³J_vic |
| 4.22 | d | 6.8 | ³J_H–H (vicinal ) |
| 6.76 / 7.65 | d,d | 8.0, 8.2 | ortho-coupled (³J_H–H) |


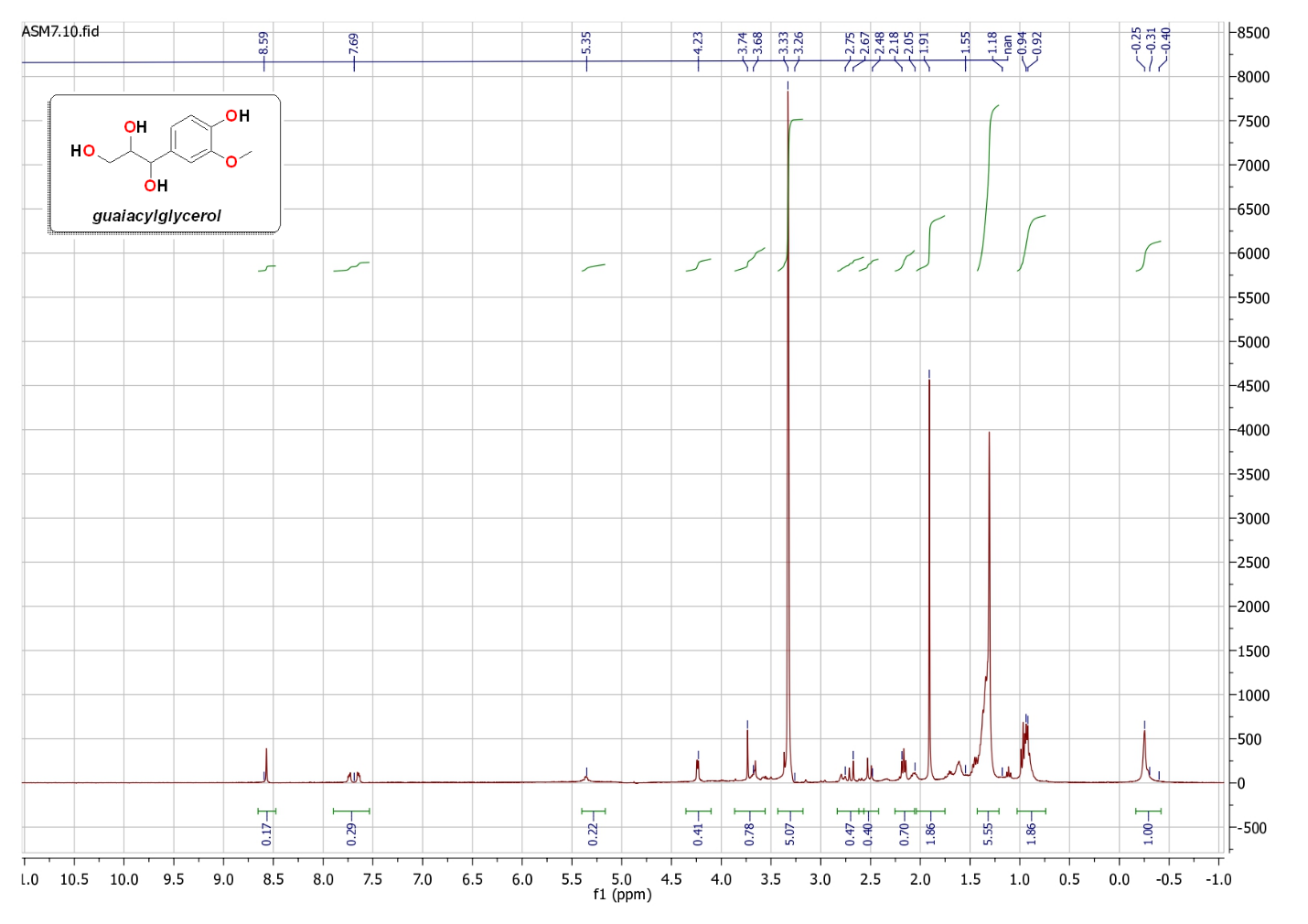


Table.S3: Compact coupling of guaiacylglycerol:

| **Proton (assignment)** | **δ (ppm)** | **Mult.** | **J (Hz)** | **Key connectivity** |
| --- | --- | --- | --- | --- |
| H-2 (Ar) | 6.90 | d | 1.8 | meta to H-6 |
| H-5 (Ar) | 6.78 | d | 8.0 | ortho to H-6 |
| H-6 (Ar) | 6.73 | dd | 8.0, 1.8 | ortho/meta from H-5/H-2 |
| HO-7 | 4.86 | d | 5.6 | ↔ H-7 |
| H-7 (CH–OH) | 4.62 | dd | 6.3, 5.6 | ↔ H-8, HO-7 |
| HO-8 | 4.40 | d | 5.3 | ↔ H-8 |
| H-8 (CH–OH) | 3.92 | dd | 7.6, 5.3 | ↔ H-7, H-9a/b |
| H-9a (CH₂OH) | 3.72 | dd | 11.5, 6.6 | ↔ H-9b, H-8 |
| H-9b (CH₂OH) | 3.57 | dd | 11.5, 5.3 | ↔ H-9a, H-8 |
| OMe | 3.83 | s | — | methoxy at C-3 |
| Ar-OH | 9.10 | s | — | phenolic OH |


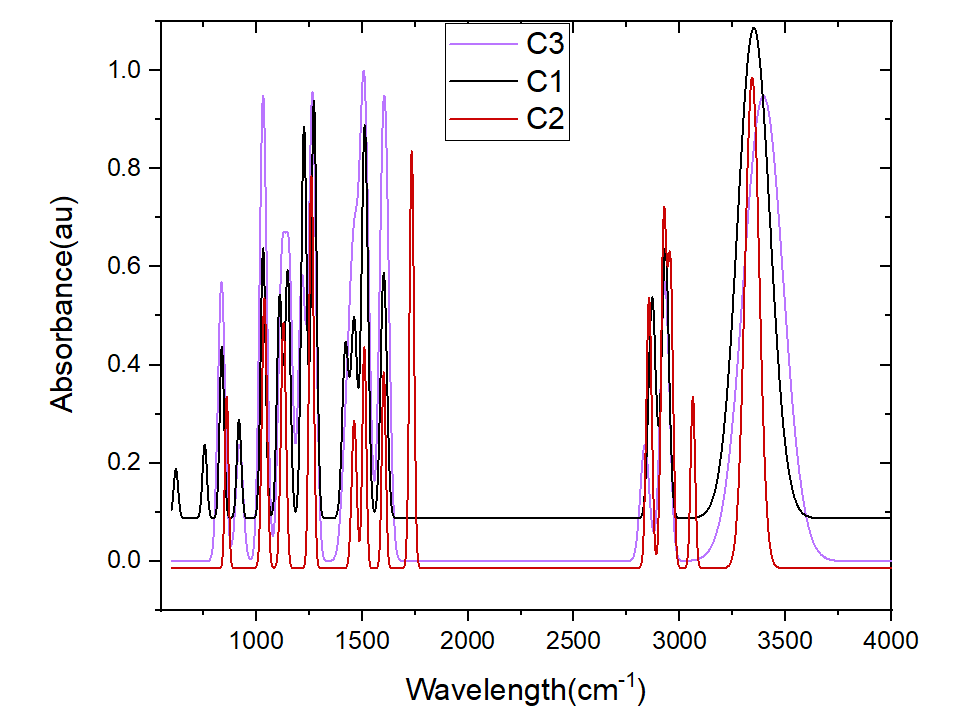


FT-IR of the compounds C1, C2, and C3

***UV analysis of C1, C2 and C3:***


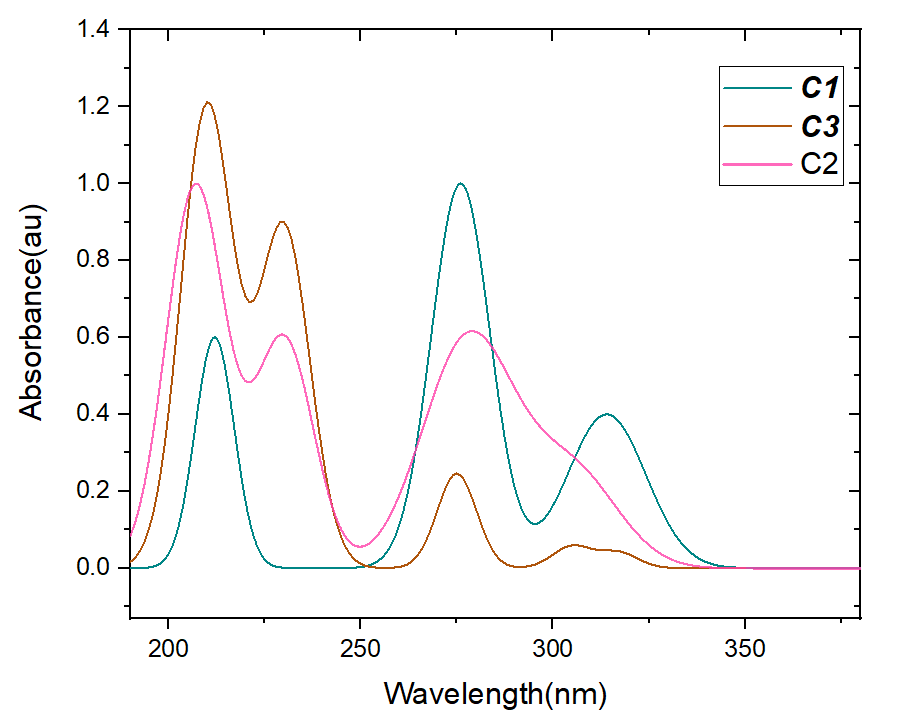


UV–Vis absorption spectra of compounds **C1**, **C2**, and **C3** recorded in methanol (1 × 10⁻⁵ M). The spectra exhibit characteristic π → π* and n → π* transitions
